# Importance of CSF-based Aβ clearance with age in humans increases with declining efficacy of blood-brain barrier/proteolytic pathways

**DOI:** 10.1038/s42003-022-03037-0

**Published:** 2022-01-27

**Authors:** Donald L. Elbert, Bruce W. Patterson, Brendan P. Lucey, Tammie L. S. Benzinger, Randall J. Bateman

**Affiliations:** 1 Department of Neurology, Dell Medical School, University of Texas at Austin, Austin, TX USA; 2 Department of Medicine, Washington University in St. Louis, St. Louis, MO USA; 3 Department of Neurology, Washington University School of Medicine, St. Louis, MO USA; 4 Hope Center for Neurological Disorders, Washington University School of Medicine, St Louis, MO USA; 5 Department of Radiology, Washington University School of Medicine, St. Louis, MO USA

**Keywords:** Computational models, Alzheimer's disease, Ageing

## Abstract

The kinetics of amyloid beta turnover within human brain is still poorly understood. We previously found a dramatic decline in the turnover of Aβ peptides in normal aging. It was not known if brain interstitial fluid/cerebrospinal fluid (ISF/CSF) fluid exchange, CSF turnover, blood-brain barrier function or proteolysis were affected by aging or the presence of β amyloid plaques. Here, we describe a non-steady state physiological model developed to decouple CSF fluid transport from other processes. Kinetic parameters were estimated using: (1) MRI-derived brain volumes, (2) stable isotope labeling kinetics (SILK) of amyloid-β peptide (Aβ), and (3) lumbar CSF Aβ concentration during SILK. Here we show that changes in blood-brain barrier transport and/or proteolysis were largely responsible for the age-related decline in Aβ turnover rates. CSF-based clearance declined modestly in normal aging but became increasingly important due to the slowing of other processes. The magnitude of CSF-based clearance was also lower than that due to blood-brain barrier function plus proteolysis. These results suggest important roles for blood-brain barrier transport and proteolytic degradation of Aβ in the development Alzheimer’s Disease in humans.

## Abstract (web summary)

To understand if brain interstitial fluid/cerebrospinal fluid (ISF/CSF) exchange, CSF turnover, blood-brain barrier function or proteolysis were affected by aging or the presence of β amyloid plaques, Elbert et al. develop a non-steady state physiological model using MRI-derived brain volumes, stable isotope labeling kinetics of Aβ, and lumbar CSF Aβ concentration. Their model suggests an important role for blood-brain barrier transport and proteolytic degradation of Aβ in the development Alzheimer’s Disease in humans.

## Introduction

Stable isotope-labeling kinetics (SILK) in humans have previously revealed that: (1) the turnover rate of Aβ_42_ (FTR42) relative to Aβ_40_ (FTR40) increases in both sporadic Alzheimer’s disease (AD) and autosomal dominant Alzheimer’s Disease (ADAD), likely due to active deposition of Aβ_42_ into plaques; (2) the production rate of Aβ_42_ relative to Aβ_40_ increases in ADAD; and (3) the turnover rates of Aβ peptides decrease with age^1–3^. One weakness of the previous analysis was the assumption that the system was at a steady state. As such, the kinetic models could not account for the effects of secretase inhibitors^4–6^, or explain the rise in the lumbar cerebrospinal fluid (CSF) concentration of Aβ peptides observed during the 36–48 h SILK studies^7–10^. Other existing non-steady-state models do not address the concentration rise observed in SILK studies^11–17^.

The goal of this approach is to more accurately model Aβ peptide kinetics in the central nervous system (CNS). Myriad processes affect Aβ turnover in the brain, including transport across the blood–brain barrier, enzymatic/lysosomal degradation, interaction and deposition into plaques, and the complex flow and exchange of CSF and interstitial fluid (ISF)^1,18–20^. Ideally, brain tissue or ISF would be sampled. However, such procedures are highly invasive^21^. Our approach was to use subject-specific MRI-derived CNS compartment volumes to better model transport of CSF from the cranial subarachnoid space (SAS) to the lumbar SAS. Although we do not directly measure or model “glymphatic” fluxes (i.e., flow within the perivascular space and brain interstitium resulting in mixing of CSF and ISF), we can infer these fluxes via the model, because the flux of Aβ transferred from ISF to CSF will equal the flux of Aβ lost from the CSF. CSF is lost from the CNS via arachnoid granulations, dural lymphatics, at the cribriform plate, and down cranial or spinal nerves^20^. These exit processes, involving bulk fluid flow of CSF, affect the Aβ concentration throughout the SAS, including at the lumbar sampling site. Via the combination of kinetic measurements, MRI volumes and CSF concentration rises upon CSF withdrawal in humans, we can estimate the turnover of Aβ in CSF and thus infer the flux of Aβ from ISF to CSF and the rate of irreversible loss of Aβ from within the ISF.

Glymphatics is defined by Nedergaard et al. as “astrocyte-mediated transport of CSF and ISF that clears metabolic waste from the interstitial space of the brain parenchyma primarily during non-REM (nonrapid eye movement) sleep and states of high slow-wave activity. This process serves a pseudolymphatic function in the CNS”^22^. Others argue that aquaporin-4 deletion does not affect dye penetration into the paravascular space and surrounding parenchyma^23^. Some have observed that transport through brain parenchyma is molecular-weight dependent suggesting a dominant role for molecular diffusion versus convective flow^17,24–27^. Correctly modeling the tortuous flow in the presence of cardiac-driven pressure fluctuations through the highly cellular cortex and dense extracellular matrix is daunting, and the current approach is desirable as it is largely if not entirely agnostic to the exact mechanism of the “glymphatic” process.

CSF flow in the CNS is generally directed from the choroid plexus to the superior sagittal sinus, although this is a simplification^28,29^. Each heartbeat and breath also induces substantial oscillatory CSF flow^30–32^. The complicated flow patterns of the CSF resulting from these pulsations are observable by MRI velocimetry and enhance mass transport in the CNS^33–35^. The presence of trabeculations and nerve roots passing through the SAS result in complicated, time-dependent recirculation patterns^15,36^. The trabeculations and nerve roots are similar to baffles, which are known in the field of reactor engineering to enhance fluid mixing^37^. Accurate flow analysis by computational fluid dynamics is impractical without higher-resolution imaging, so the model presented here assumes that oscillatory CSF flow results in the perfect mixing of fluids within distinct compartments in the SAS. This simplification is consistent with our previous compartmental model that fit the SILK data exceptionally well^1,2^. The model accounts for changes in lumbar space volume due to the hourly withdrawal of CSF from the lumbar space and potential CSF leaks due to the indwelling catheter, which is perhaps the most important feature of the model.

It is believed that the rate of nucleation and growth of amyloid plaques depends upon the concentration of Aβ_42_ in the cortical interstitial fluid^12,38^. Thus, changes in the rate of clearance of Aβ_42_ from the cortical interstitial fluid may be a critical factor in the onset of plaque formation, just as differences in the rate of production of Aβ_42_ affect the age of onset of Alzheimer’s disease^1^. In this study, we estimate the rates of clearance of Aβ in cortical interstitial fluid due to different processes, such as transport across the blood–brain barrier, proteolysis, and deposition into plaques. Transport of Aβ_42_ across the blood–brain barrier is mediated by lipoprotein-related protein 1 (LRP-1)^39^, leading to the transfer of Aβ_42_ from the brain interstitial fluid to the blood^40^. Binding of Aβ_40_ and Aβ_42_ to LRP-1 is saturable and clearance across mouse brain capillaries is blocked by antibodies against LRP-1^41^. Expression of LRP-1 declines in aging rats and is negatively correlated with the concentration of Aβ_40_ and Aβ_42_ in rat brain cortex homogenates^42^. Multiple enzymes contribute to the proteolysis of Aβ_42_ within the CNS, including secreted and membrane-bound enzymes such as an angiotensin-converting enzyme, neprilysin, insulin-degrading enzyme and various MMPs, in addition to degradation downstream of endocytosis or phagocytosis by cells, particularly microglia and monocyte-derived macrophages^43^. Transport across the BBB, enzymatic degradation and deposition into plaques within the brain will result in similar kinetics for isotope-labeled Aβ, as all processes likely exhibit close to first-order kinetics and occur within the same compartment. In contrast, transfer of Aβ from ISF to CSF will result in elimination of Aβ by processes governed by CSF turnover kinetics. CSF-based processes will affect the shape of the SILK curves differently from processes that occur within the ISF, and this provides the rationale for the current study.

## Results and discussion

### Correlation of MRI-derived volumes with age and steady-state model parameters

CNS volumes were recorded by MRI for 100 study subjects who also completed SILK kinetic studies, with four subjects excluded due to poor fits of the model to SILK or lumbar concentration data. All of the subjects were part of earlier published studies^1,2^. Subject demographics, lumbar CSF tau and Aβ concentrations, and results from the previous steady-state model are shown in Supplementary Tables 1 and 2. Correlations of MRI-measured CNS volumes with amyloid status were consistent with findings in the literature (Supplementary Table 3)^44–47^. Amygdala, hippocampus, and precuneus volumes declined most dramatically due to β-amyloidosis. Total ventricle volume was increased in amyloid-positive subjects, while the total gray volume decreased. The effects of normal aging on CNS volumes and thicknesses were assessed in amyloid-negative subjects to eliminate effects of β-amyloidosis (Supplementary Table 4). Putamen, accumbens-area, thalamus, and hippocampus volumes declined most dramatically with age, as expected.

### Development of a subject-specific physiological model

**Fig. 1:**
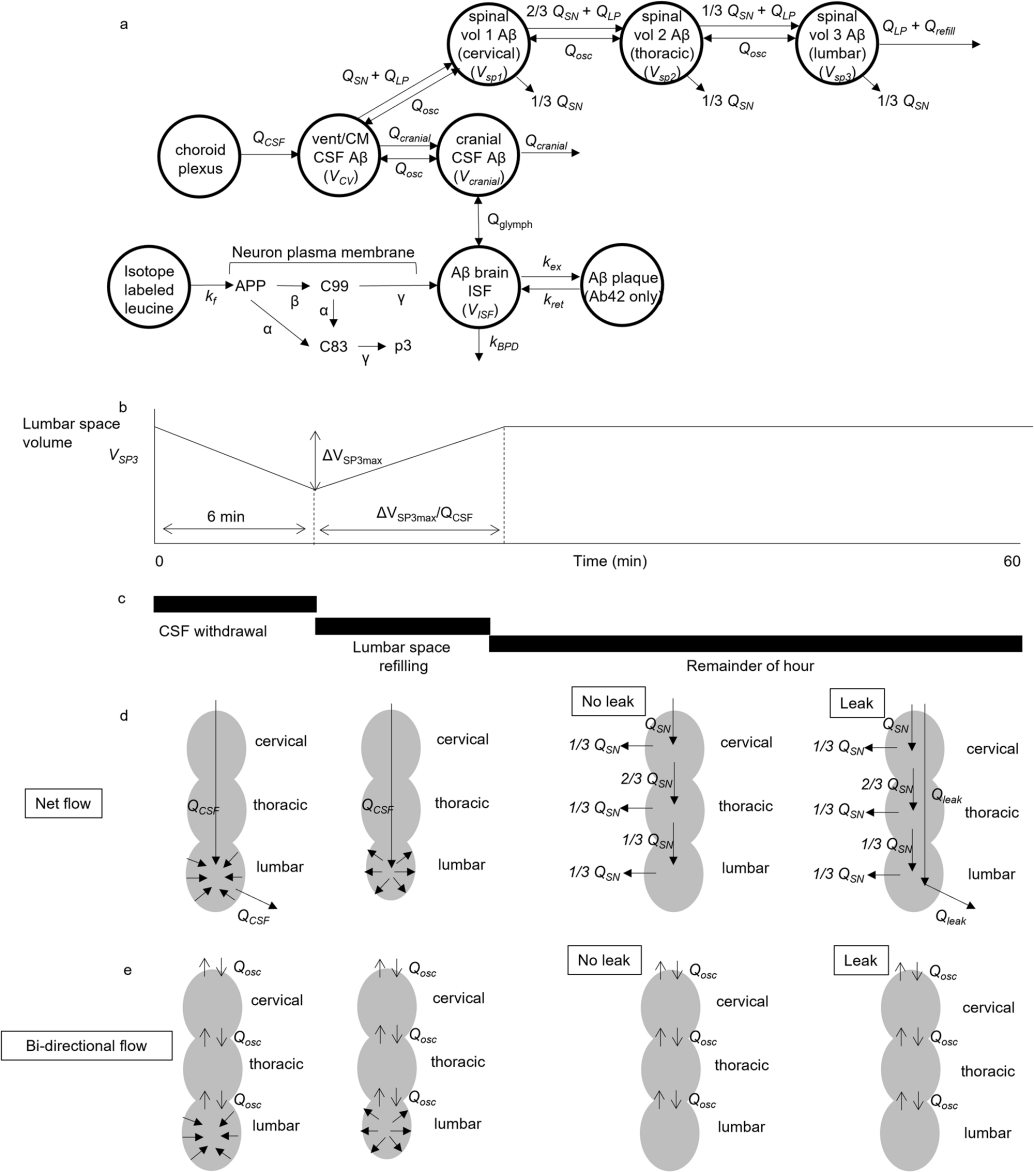
Model of Aβ production, transport, and clearance. Physiological model of CNS during SILK study, incorporating fluid flows, ISF and CSF compartment volumes, and Michaelis–Menten kinetics for Aβ production. **a** Model structure. **b** Flows in the spinal SAS due to hourly CSF withdrawal. The volume of the lumbar SAS decreases when the CSF withdrawal rate *Q*_*LP*_ exceeds the CSF production rate (Q_CSF_) and subsequently refills with CSF to its original volume. **c** Timeline of hourly CSF withdrawal. **d** Net flow in the spinal SAS. **e** Bidirectional flow in the spinal SAS.

A new physiological model was developed to decouple transport of Aβ peptides within CSF from (1) transport across the blood–brain barrier, (2) changes in proteolytic degradation, and (3) deposition into plaques. The new physiological model (Fig. 1a) assumed that the CNS consists of the following compartments: (1) cells producing Aβ via enzymatic reactions at their plasma membranes following Michaelis–Menten kinetics, (2) brain ISF, (3) cranial subarachnoid space (SAS), (4) cisternal and ventricular SAS, and (5) spinal SAS divided into cervical, thoracic and lumbar regions. The model was written as a nonlinear system of 51 ordinary differential equations (Supplementary Methods 1). Parameter optimization and differential equation solving were performed in Julia^48–50^. The volumes of compartments were estimated from Freesurfer analysis of MRI scans (Supplementary Methods 2). The spinal volumes were not measured in this study and literature values were assumed for the spinal SAS volumes (*V*_*SP1*_ ≈ cervical SAS, *V*_*SP2*_ ≈ thoracic SAS, *V*_*SP3*_ ≈ lumbar SAS)^51–53^. In addition, a range of CSF volumes was examined to discover the best fit to the observed change in lumbar concentration of Aβ during the course of the 36–48 h SILK experiment. Transfer between compartments was modeled as volumetric flows (mL/h), which were divided by the volumes of the compartments to obtain first-order rate constants for mass transfer between compartments.

CSF production was assumed to entirely arise from the choroid plexus, specified as the CSF production rate *Q*_*CSF*_. During the SILK study, 6 mL of CSF were withdrawn every hour from an indwelling lumbar catheter. The withdrawal occurred over 5–10 min. During the hourly CSF collection, the CSF withdrawal rate (*Q*_*LP*_) was much greater than the model-predicted CSF production rate (*Q*_*CSF*_) in all subjects. It was thus assumed that the volume of the lumbar space (*V*_*SP3*_) decreased during withdrawal (Fig. 1b–e), which has been observed in humans by MRI during lumbar puncture^51^. After CSF withdrawal, CSF production served to refill the lumbar space to its original volume. Although the majority of CSF was assumed to be reabsorbed within the cranial compartment, some amount of CSF was assumed to be lost down spinal nerves (*Q*_*SN*_)^54^. *Q*_*osc*_ and *Q*_*glymph*_ are bidirectional continuous flows that approximate the oscillatory flows due to the cardiac and respiratory cycles. For some subjects with large lumbar CSF Aβ concentration rises, the data were better fit assuming a leak due to catheterization (*Q*_*leak*_). Further details of the model are provided in Supplementary Methods 1.

**Fig. 2:**
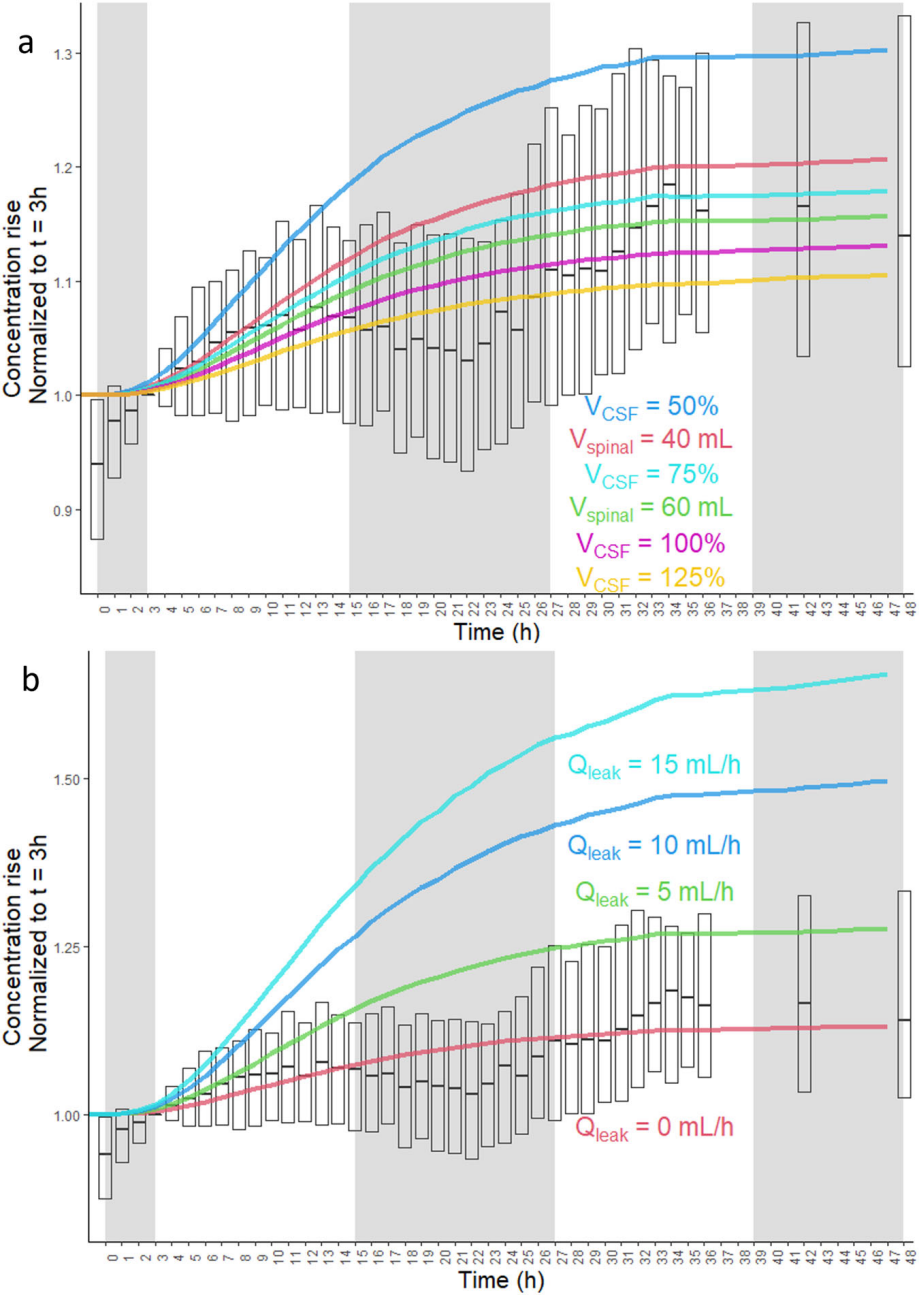
Increasing lumbar Aβ CSF concentration due to hourly CSF withdrawal. Normalized lumbar CSF concentrations of Aβ peptides averaged over all subjects (*n* = 96). Lumbar CSF Aβ concentration time courses for each subject were first normalized to time = 3 h to account for the sleep effect from the previous night (experiments started at 8 a.m.). The Aβ concentration was the average of Aβ_38_, Aβ_40_, and total Aβ for each subject (total Aβ was from an immunoprecipitation with an antibody recognizing a sequence common to Aβ_38_, Aβ_40_, and Aβ_42_). Some subjects (*n* = 17) only had concentration timecourse measured by ELISA for Aβ_40_ and Aβ_42_. The ELISA time courses were averaged for these two peptides at each time point. **a** The total volume of CSF (*V*_*CSF*_) was scaled between 50 and 125% in separate simulations, and the volume of spinal CSF was varied from 40 to 80 mL. **b** The presence of a CSF leak around the catheter was also simulated, ranging from 0 to 20 mL/h, or equal to the entire CSF production rate (*Q*_*leak*_ = *Q*_*CSF*_). Medians and means of measurements are shown, and bars represent the interquartile range. Shaded areas represent the sleep effect. Simulations of CSF leaks equal to 20 mL/h or *Q*_*CSF*_ overlapped with *Q*_*leak*_ = 15 mL/h. For the full boxplot of measured concentrations, see Supplementary Fig. 1.

The lumbar CSF concentration rise of Aβ peptides was substantial (Fig. 2 and Supplementary Fig. 1), with a mean rise of 27% between 0 and 36 h. All experiments began at 8 a.m. (time = 0) and a dip in the lumbar concentration of Aβ peptides was seen during the nighttime and following morning, which has been previously associated with the effects of sleep^9,10^. However, sleep was not modeled in this study as the sleep-wake cycle was not controlled and was not apparent in 19 out of 96 subjects. In this study, the subjects arose early (how early was uncontrolled) to reach the hospital by 7 a.m. and the study was started at 8 a.m. They were instructed to remain mostly in bed for 36–48 h and could sleep/nap at will. Of the 77 subjects that showed a sleep effect, many showed abbreviated dips, double dips, or prolonged dips in the lumbar Aβ concentration. SILK and concentration-time courses for all 96 subjects are presented in Supplementary Data 3. To reduce the effect of sleep on the conclusions, concentration data was not fit to the model between 11 p.m. and 11 a.m., accounting for transport delays between ISF and the lumbar space. The sleep effect also overlapped with the first three hours of the experiment.

**Fig. 3:**
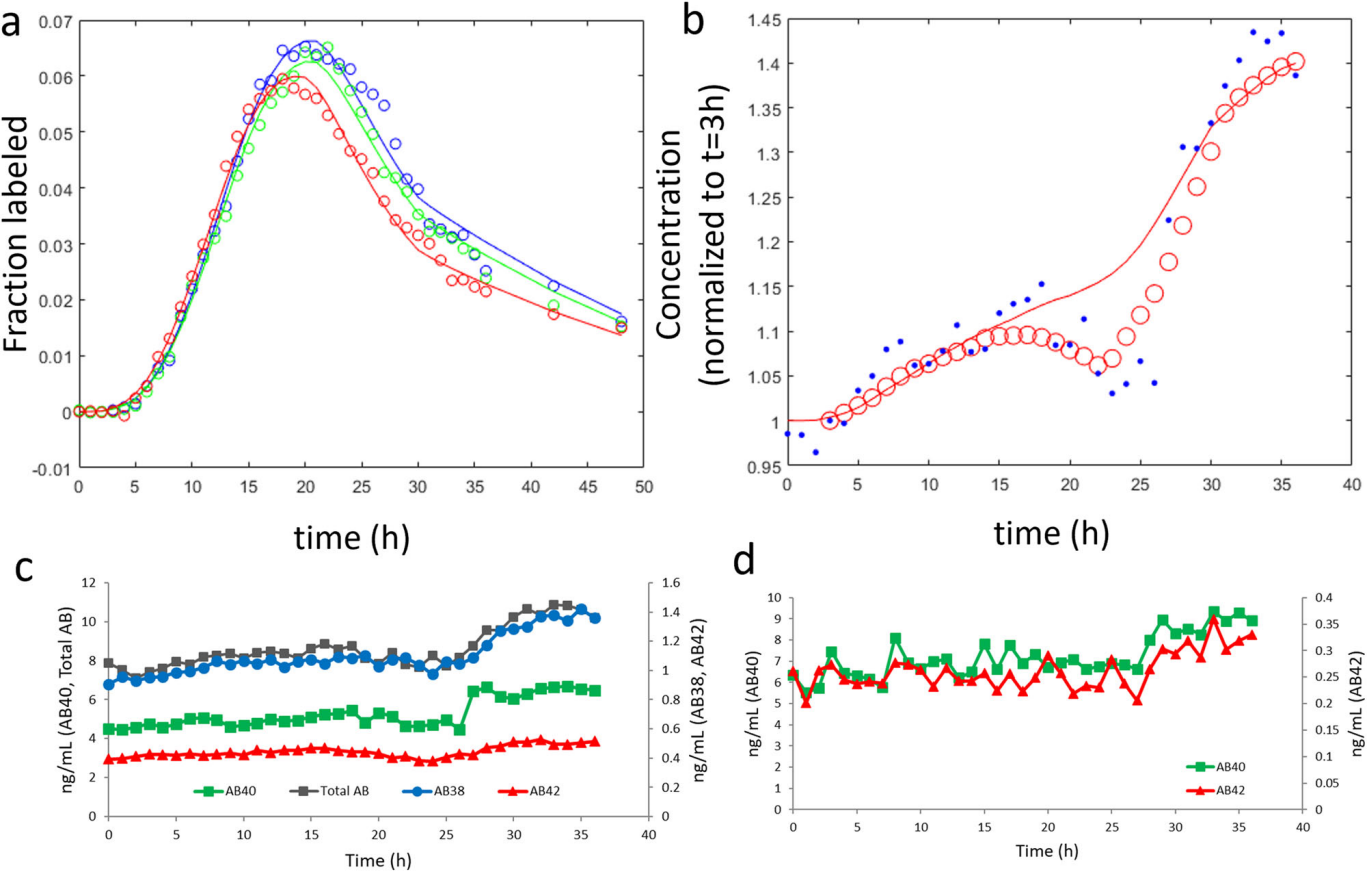
Example of model fits to SILK and lumbar Aβ concentration. The physiological model fits with step changes in *Q*_*leak*_ at 3 h and 20 h for a single amyloid-positive subject. **a** Fit of the physiological model to SILK data. Blue = Aβ_38_, green = Aβ_40_, red = Aβ_42_. **b** Fit of lumbar CSF concentration data (0 < time < 3, *Q*_*leak*_ = 8 mL/h; 3 < time ≤ 20 h, *Q*_*leak*_ = 0 mL/h; 20 ≤ time < 30 h, *Q*_*leak*_ = 13 mL/h; time ≥ 30 h, *Q*_*leak*_ = 0 mL/h). Blue circles = mass spectrometric measurements normalized to time = 3 h. Red circles = moving mean smoothed data. **c** Lumbar CSF concentrations were measured by immunoprecipitation followed by quantitative mass spectrometry, showing the abrupt change in concentration at about 27 h for all four peptides measured. **d** Lumbar CSF concentrations measured by ELISA also showed an abrupt change at about 27 h for both peptides measured.

The physiological model predicted a lumbar Aβ concentration rise of 12% between 3 and 36 h (see *V*_*CSF*_ 100% curve in Fig. 2a and *Q*_*leak*_ = 0 mL/h curve in Fig. 2b). This was lower than both the mean and median concentration rises between 3 and 36 h (19% and 17%, respectively). The total CSF volume and/or the spinal CSF volume were thus systematically varied to improve the fit of the model to the observed concentration rise, selecting the simulation with the lowest sum-of-squares residual. For 20 out of 96 subjects, the concentration rise was greater than that predicted even if the total CSF volume was decreased by 50%. For these subjects, an additional parameter was added, *Q*_*leak*_, which represented continual leakage of CSF around the indwelling catheter. A leak was also assumed for the seven subjects with clinical records of a blood patch to seal a CSF leak. *Q*_*leak*_ was not different by amyloid status but did significantly decrease with age (Table 1 and Supplementary Table 5). For ten of the subjects, the leak rate appeared to change at specific points during the 36–48 h experiment, and *Q*_*leak*_ was allowed to change to better fit the data (for example, see Fig. 3). Fits to SILK and lumbar concentration data for all 96 subjects are included as Supplementary Data 3.

**Table 1:** Model results.

		Predicted marginal means (S.E.) at age = 69.9 y			Correlation coefficient with age	
		Amyloid negative (*N* = 58)	Amyloid positive (*N* = 38)	*P* value^a^	Amyloid negative (*N* = 58)	*P* value
CSF leak	*Q*_*leak*_ > *0?*	20/58	10/38	0.40^*^		
	*Q*_*leak*_ (mL/h)	2.6 (0.6)	2.6 (0.8)	0.92	−0.36	***0.0050***
Aβ clearance	*k*_*BPD38*_ (h^−1^)	0.31 (0.02)	0.22 (0.03)	*0.015*	−0.68	***<0.0001***
	*k*_*BPD40*_ (h^−1^)	0.37 (0.03)	0.24 (0.03)	***0.0024***	−0.59	***<0.0001***
	*k*_*BPD42*_ (h^−1^)	0.53 (0.06)	0.96 (0.07)	***<0.0001***	−0.27	*0.044*
	*k*_*BPD42*_*/k*_*BPD38*_	2.1 (0.4)	5.3 (0.5)	***<0.0001***	0.24	0.069
	*k*_*BPD42*_*/k*_*BPD40*_	1.7 (0.3)	4.8 (0.4)	***<0.0001***	0.13	0.33
	*k*_*BPD40*_*/k*_*BPD38*_	1.36 (0.08)	1.1 (0.1)	0.10	0.21	0.11
Aβ production (w/o mutation carriers)	*V*_*max,γ42*_*/V*_*max,γ38*_	1.02 (0.04)^b^	1.07 (0.06)^c^	0.52	0.28^b^	0.039
	*V*_*max,γ42*_ (μg/(mL·h))	125 (5)^b^	139 (8)^c^	0.15	0.34^b^	0.013
APP production (w/o mutation carriers)	*k*_*f*_ (ng/h)	3500 (300)^b^	3400 (500)^c^	0.79	−0.58^b^	<0.0001
	Total gray volume (mL)	590 (8)^b^	570 (10)^c^	0.15	−0.42^b^	0.0014
Exchange	*k*_*ex42*_ (h^−1^)	0.06 (0.02)	0.17 (0.02)	***<0.0001***	0.087	0.52
CSF fluid flow	*Q*_*CSF*_ *=* *Q*_*glymph*_ (mL/h)	23 (1)	26 (2)	0.31	−0.0062	0.99
	*Q*_*osc*_ (mL/h)	8.8 (0.8)	11 (1)	0.16	0.47	***<0.0001***
	[Aβ_40_]_ISF_/[Aβ_40_]_lumbar_	10.1 (0.8)	10 (1)	0.85	−0.51	***<0.0001***
	Predicted cisternography half-life (h)	20.7 (0.6)	19.8 (0.7)	0.32	−0.30	*0.021*
	*V*_*CSF*_ (mL)	300 (10)	310 (10)	0.47	0.38	***0.0030***
*Flux Aβ*_38_	CSF-based (ng/min)	3.1 (0.4)	3.4 (0.5)	0.54	−0.24	0.070
	BBB + proteolysis (ng/min)	4.9 (0.4)	3.2 (0.5)	***0.0082***	−0.81	***<0.0001***
	% CSF-based	43 (3)	52 (3)	*0.037*	0.47	***<0.0001***
*Flux Aβ*_*40*_	CSF-based (ng/min)	14 (2)	17 (2)	0.29	−0.33	*0.011*
	BBB + proteolysis (ng/min)	28 (2)	16 (3)	***0.0037***	−0.76	***<0.0001***
	% CSF-based	39 (2)	50 (3)	***0.0042***	0.45	***<0.0001***
*Flux Aβ*_*42*_	CSF-based (ng/min)	2.2 (0.2)	1.7 (0.3)	0.19	−0.30	*0.021*
	BBB + proteolysis (ng/min)	4.1 (0.4)	1.6 (0.5)	***<0.0001***	−0.70	***<0.0001***
	Deposition (ng/min)	1.4 (0.4)	4.6 (0.5)	***<0.0001***	−0.21	0.11
	% CSF-based	33 (2)	22 (3)	***0.0014***	0.33	*0.012*

^*^Chi-square statistic = 0.71.

^a^*t* test of predicted marginal means (least-squares means) except where noted by *. See Supplementary Table 5 for interactions between age and amyloid status; italic values signify: *P* < 0.05; bold italic values signify: *P* < 0.01.

^b^*N* = 54.

^c^*N* = 33.

### Kinetic results from the physiological model

**Fig. 4:**
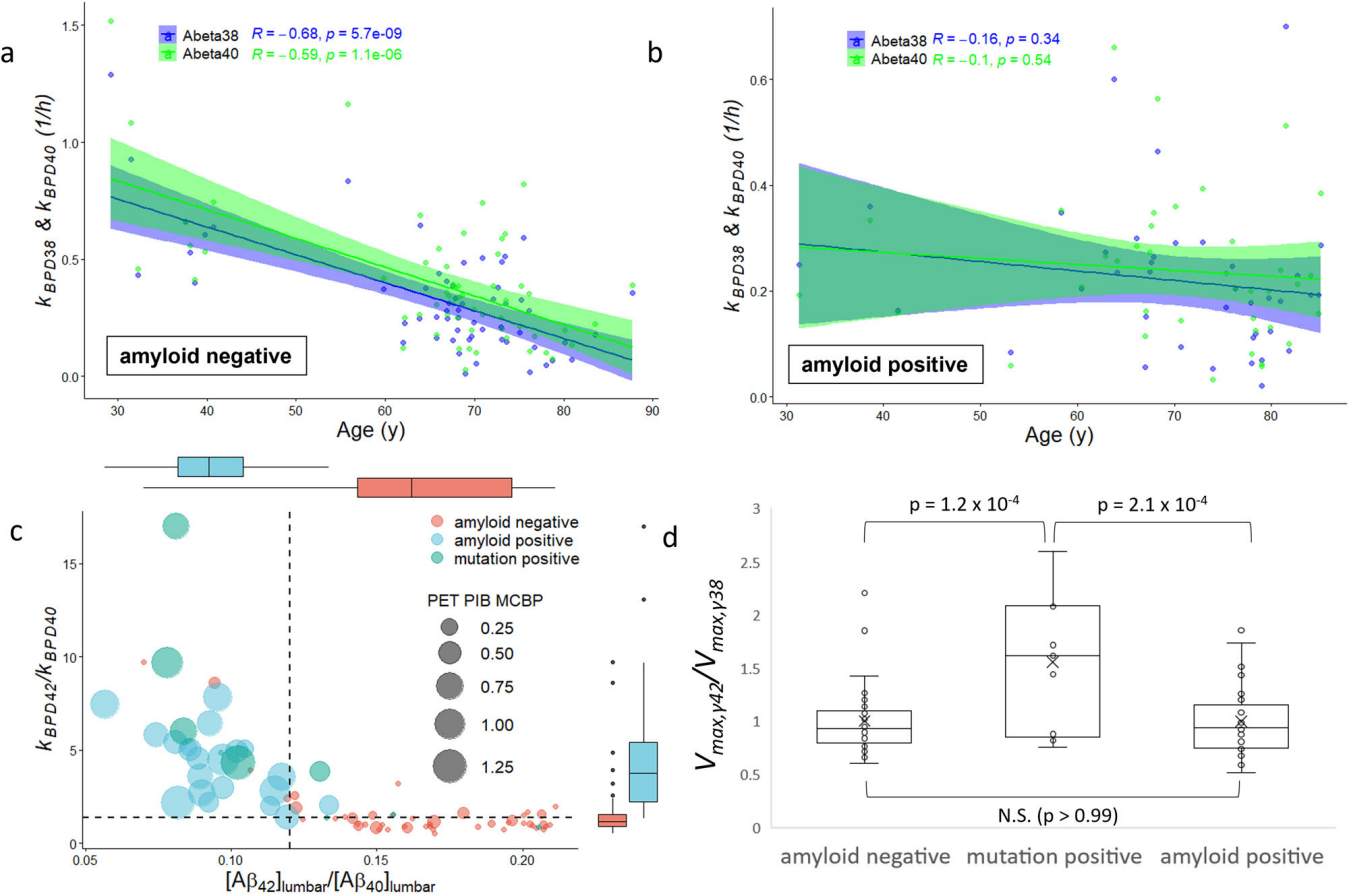
Derived Aβ production and clearance rate constants. Aβ clearance and production rate constants. **a** The rate constant describing the irreversible loss of Aβ peptides from the brain ISF compartment (*k*_*BPD*_) significantly declined with age in amyloid-negative subjects for Aβ_38_ and Aβ_40_ (*n* = 58; also see Table 1). **b** The age effect on *k*_*BPD38*_ and *k*_*BPD40*_ was absent in amyloid-positive subjects (*n* = 38). **c** The rate of irreversible loss of Aβ_42_ (*k*_*BPD42*_) was elevated relative to Aβ_40_ (*k*_*BPD40*_) in the presence of amyloid plaques, as evidenced by elevated PET PIB MCBP (*n* =  77). This elevation likely represented active deposition of Aβ_42_ into plaques, and a cutoff of 1.3 (horizontal dashed line) distinguishes most plaque-bearing subjects from non-plaque-bearing subjects. Declining ratios of lumbar CSF Aβ_42_ to Aβ_40_ are a hallmark of AD, with a cutoff of 0.12 in common use (vertical dashed line). See statistical comparisons in Table 1. **d** The gamma-secretase production rate constant ratio *V*_*max,γ42*_/*V*_*max,γ38*_ reflects the kinetics of gamma-secretase toward the production of Aβ_42_ relative to Aβ_38_. The production rate ratio did not differ by amyloid status, but presenilin mutation carriers had elevated production of Aβ_42_ relative to Aβ_38_ as compared to both groups of non-mutation carriers. ANOVA with Tukey post hoc analysis.

The parameter *k*_*BPD*_ describes irreversible loss in the ISF compartment by processes that include: (1) transport across the blood–brain barrier (“B”), (2) proteolytic degradation (“P”), and (3) permanent deposition into plaques (“D”). *k*_*BPD*_ is similar to FTR from the steady-state model, but the effects of CSF transport are modeled explicitly, which should allow *k*_*BPD*_ to more accurately represent processes that occur in the brain ISF. Like FTR, both *k*_*BPD38*_ and *k*_*BPD40*_ were highly correlated with age in amyloid-negative subjects (Table 1 and Fig. 4a). Unlike FTR, *k*_*BPD38*_ and *k*_*BPD40*_ are not correlated with age in the amyloid-positive subject (Fig. 4b). However, the CSF production rate (*Q*_*CSF*_) was negatively correlated with age in amyloid-positive subjects (*r* = −0.42, *P* = 0.0079, *n* = 38) but not in amyloid-negative subjects (*r* = −0.0062, *P* = 0.96, *n* = 58). This implies that changes in Aβ clearance in normal aging are driven by changes in blood–brain barrier transport or proteolysis (deposition should be minimal in normal aging). Aβ clearance in amyloid-positive subjects appears to be influenced by changes in CSF production, and potentially from the substantial decrease in brain parenchymal volumes and increase in ventricle volume (Supplementary Table 3).

*k*_*BPD38*_ and *k*_*BPD40*_ are significantly lower in amyloid-positive subjects after controlling for age (Table 1). However, *k*_*BPD42*_ is significantly *higher* in amyloid-positive subjects, as is the ratio *k*_*BPD42*_/*k*_*BPD40*_ (Table 1 and Fig. 4c). This mirrors the finding from the steady-state model that the turnover of Aβ_42_ was elevated in amyloid-positive subjects relative to Aβ_40_. Because transport and proteolysis rates of Aβ_40_ and Aβ_42_ are expected to be similar, the increased clearance of Aβ_42_ is believed to represent deposition of Aβ_42_ into plaques^1,2^. The ratio *k*_*BPD42*_/*k*_*BPD40*_ is not correlated with age in amyloid-negative subjects (Table 1) or in amyloid-positive subjects (*r* = −0.29, *P* = 0.073, *n* = 38).

The forward rate constant for reversible exchange in the ISF compartment (*k*_*ex42*_) was significantly higher in amyloid-positive subjects (Table 1). It is hypothesized that soluble Aβ_42_ becomes temporarily insoluble sometime between production and clearance. The greater magnitude in subjects with AD implies a role for plaques in this process^2^. Isotope labeling of plaques has also been observed in SILK subjects postmortem^55^.

With certain presenilin mutations, the rate of production of Aβ_42_ is expected to be elevated relative to the rate of production of Aβ_40_, which was indeed observed with the steady-state model^1^. However, the steady-state model also exhibited a strong correlation between the production rate ratio of Aβ_42_ to Aβ_40_ and the lumbar CSF concentration ratio [Aβ_42_]_lumbar_ /[Aβ_40_]_lumbar_ (Supplementary Fig. 2a). This would require a highly specific change in the kinetics of secretases in AD, or changes in intracellular trafficking that selectively decreased the rate of production of Aβ_42_ relative to Aβ_40_, which both seem unlikely. In the physiological model, the *V*_*max*_ of gamma-secretase for the production of Aβ_42_ (*V*_*max,γ42*_) was determined relative to *V*_*max,γ40*_, with *V*_*max,γ40*_ fixed for all subjects to a literature-derived value^56,57^. *V*_*max,γ42*_ was thus normalized by *V*_*max,γ38*_ instead of *V*_*max,γ40*_. The production rate ratio of Aβ_42_ relative to Aβ_38_ by gamma-secretase was elevated in presenilin mutation carriers compared to amyloid negatives and amyloid positives (Fig. 4d). There was no difference in the relative production rate based on amyloid status, as expected, and in contrast to the steady-state model (Supplementary Fig. 2b). Without normalization, *V*_*max,γ42*_ was higher in mutation positives versus amyloid negatives as expected, but the comparison with amyloid positives did not reach significance (Supplementary Fig. 2c).

The production rate of APP (*k*_*f*_) declined with age, perhaps related to the decline in total gray volume (Table 1) and a loss of synaptic activity^58–60^. The production rate of APP was also compared between presenilin mutation carriers and sibling non-mutation-carrier controls (Table 2). In these age-matched subjects, the production rate of Aβ_42_ increased due to the presence of presenilin mutations, but *k*_*f*_ was not different.

**Table 2:** Production rates in presenilin mutation carriers and age-matched non-carriers.

	Mutation-carrier mean (S.E.) *N* = 9	Sibling non-carriers mean (S.E.) *N* = 10	*P* value^a^
Age	45 (4)	49 (5)	0.61
*k*_*f*_ (ng/h)	8000 (1000)	8000 (1000)	0.99
*V*_*max,γ42*_ (μg/(mL h))	1.6 (0.2)	0.80 (0.05)	***0.0079***
*V*_*max,γ42*_*/ V*_*max,γ38*_	160 (20)	85 (3)	***0.0014***
Total gray volume (mL)	600 (20)	640 (10)	0.14

^a^*t* test; values in italics signify *P* < 0.05; values in bold italic signify: *P* < 0.01.

The CSF production rate *Q*_*CSF*_ should be identical to the CSF production rate typically measured by, for example, the modified Masserman method^61^. *Q*_*CSF*_ did not differ by amyloid status (Table 1). The mean values were similar to those found in a carefully performed study in which the CSF production rate was found to be 24.0 ± 5.00 mL/h (95% CI: 16.2–33.8 mL/h)^62^. The CSF production rate has been found to decline with age^61^. *Q*_*CSF*_ did not vary significantly with age in amyloid-negative subjects (Table 1). CSF pressure transients and thus CSF production rate were not measured during the data collection and the CSF production rate is inferred from the data. Our mode of measurement is not as precise and the decrease is not detected. However, on average, the CSF production rates are reasonable and arise from the model structure and data without being fit. In addition, amyloid subjects under 60 years in age show a greater CSF production rate than those over 60, but it is not significant (*P* = 0.2; Supplementary Table 6). However, *Q*_*CSF*_ did decline with age in amyloid-positive subjects, as mentioned previously.

The physiological model accounts for the oscillatory flow of CSF driven by circulatory and respiratory pulsations. The oscillatory flow was modeled as a continuous bidirectional flow with magnitude *Q*_*osc*_. *Q*_*osc*_ did not differ between AD and non-AD subjects (Table 1). *Q*_*osc*_ increased with age, while the ISF-to-lumbar concentration “gradient” [Aβ_40_]_ISF_/[Aβ_40_]_lumbar_ decreased with age (Table 1), consistent with previous observations in humans^61^. The concentration gradient is highly positively correlated with 1/*Q*_*osc*_^1/2^ (*r* = 0.82, *P* = 1.3 × 10^−24^, *n* = 96). *Q*_*osc*_ is mathematically similar to a diffusion coefficient and higher values will tend to decrease the ISF/lumbar concentration gradient. It is known that the total cerebral blood flow decreases in aging, as does the cervical CSF stroke volume^63^. This suggests that the enhanced mixing of CSF that decreases the ISF/lumbar gradient in aging is due to biomechanical factors other than CSF pulsatility. The increase in *Q*_*osc*_ with age may be similar to the increase in pulse wave velocity with age due to stiffening of the arteries^64^. Although ISF-to-lumbar gradients can be inferred from experiments, there is scant evidence of a ventriculo-lumbar gradient for Aβ^21,65^, in contrast to the negative gradient for brain proteins such as tau and the positive gradient for blood proteins such as albumin^66^.

Allowing bidirectional flow results in mass transfer down concentration gradients. For example, within the model, the introduction of a labeled species in the lumbar space results in eventual transport of the label to the brain, similar to cisternograpy and related MRI-based CNS tracer studies^54,67,68^. Although the study protocol did not include cisternography, the model makes specific predictions about the distribution and disappearance of label within the CNS. This includes a prediction of a “cisternography half-life”, the half-life in the CNS of a labeled compound introduced into the lumbar space. Moriyama et al. studied the effects of age on cisternography half-life and developed a linear regression model^67^. The Moriyama et al. model predicted a mean cisternography half-life for the subjects in our study of 20 ± 2.4 h, compared to 21 ± 4.5 h predicted by the physiological model. The predicted cisternography half-life was strongly influenced by the exit of CSF from the CNS down spinal nerves and this was used to fix *Q*_*SN*_ at 10% of *Q*_*CSF*_ for all subjects. Higher or lower values of *Q*_*SN*_ resulted in unreasonable values for the predicted cisternography half-life (Supplementary Fig. 3). The chosen value for *Q*_*SN*_ was slightly lower than previous estimates (12–25% of *Q*_*CSF*_)^69^. Although the observed increase in *Q*_*osc*_ with age would tend to decrease the cisternography half-life, the increase in the total volume of CSF (*V*_*CSF*_) with age seems to negate this effect (Table 1). While cisternography data is useful for imaging tracer in the entire CNS simultaneously, modern methods such as glymphatic MRI can contribute more refined spatial information that will be useful in the development of future versions of the model with more accurate geometric modeling of the CNS^70–72^.

### Flux of Aβ through distinct pathways

The concentrations and fluxes of Aβ peptides within ISF are difficult to measure in humans. The measured Aβ_40_ concentration in homogenized cortex varies widely but tends to be in the range of 1–10 ng/g wet cortical tissue^40,73–75^, although some studies report much higher values^76–78^. APP is an integral membrane protein and Aβ peptides are predominantly extracellular^79^ (particularly when considering endosomes and lysosomes to be topologically extracellular). Assuming a cellularity of the cortex of ~90%^80^, this suggests that cortical ISF concentrations are about 10× higher than concentrations measured in the homogenized cortex. The physiological model specifically incorporates the volume of gray matter ISF, and the mean rise in Aβ_40_ concentration from lumbar SAS to brain ISF ([Aβ_40_]_ISF_/[Aβ_40_]_lumbar_) was about tenfold and did not differ by amyloid status (Table 1). However, the concentration of Aβ in homogenized cortex should be about tenfold lower than the concentration in brain ISF. Using this assumption, the physiological model predicted a concentration of Aβ_40_ in the homogenized cortex of 6.6 ± 3.5 ng/g wet tissue, within the range of typical values (assuming a density of the cortex tissue of 1.03 g/mL^81^). However, using microdialysis, the concentration of Aβ in the brain ISF is about the same as in ventricular and lumbar CSF, and this is more than an order of magnitude lower than the value predicted by the physiological model^21,82^. Given the dramatic concentration rise observed during sustained withdrawal of lumbar CSF, some fluid within the CNS must have a higher concentration of Aβ and this is likely the ISF. This suggests that microdialysis measurements underreport the ISF concentration of Aβ.

The flux of Aβ_40_ peptides from the CNS into blood was previously reported as 9.7 ng/min, which was found by measuring the venous–arterial Aβ concentration difference during inferior petrosal sinus sampling^40^. This flux should include Aβ transported to the venous system by CSF absorption at the superior sagittal sinus *and* Aβ transported across the blood–brain barrier. It should not include Aβ transported to the lymphatic system via CSF absorption at the dura, cribriform plate, or via perivascular transport^20^. The physiological model predicted a mean Aβ_40_ flux of 15 ± 13 ng/min due to CSF absorption in all subjects according to Eq. (1).

1 $$Flux\,of\,A{\beta }_{40}\,by\,CSF\,absorption={Q}_{crainal} \times {[A{\beta }_{40}]}_{cranial}+{Q}_{spinalnerve}\times{[A{\beta }_{40}]}_{spinal}$$

A key feature of glymphatics is the entrance of CSF along periarterial pathways and ISF exit along perivenous pathways^83^. The flux of Aβ_40_ peptides from the brain ISF into the CSF is given by Eq. (2). Due to mass balance, Eqs. (1) and (2) must be equal at steady state or pseudo-steady state.

2 $$Flux\,of\,A{\beta }_{40}\,from\,ISF\,to\,CSF={Q}_{glymph}\times({[A{\beta }_{40}]}_{ISF}-{[A{\beta }_{40}]}_{cranial})$$

The mean rate of irreversible loss of Aβ_40_ peptides by transport across the BBB or via proteolysis is predicted by the physiological model to be 25 ± 28 ng/min (Eq. (3)). This is greater than the experimentally measured value from inferior petrosal sinus sampling, suggesting that at least 60% of Aβ_40_ is cleared from the brain by proteolysis. The amount of CSF that is absorbed at arachnoid granulations is controversial and may be quite low^84^. In that case, the vast majority of Aβ measured during inferior petrosal sinus sampling would represent transport across the blood–brain barrier.

3 $$Flux\,of\,A{\beta }_{40}\,via\,BBB\,transport,\,proteolysis\,and\,deposition={k}_{BPD40}\times{[A\beta 40]}_{brain}x{V}_{brainISF}$$

**Fig. 5:**
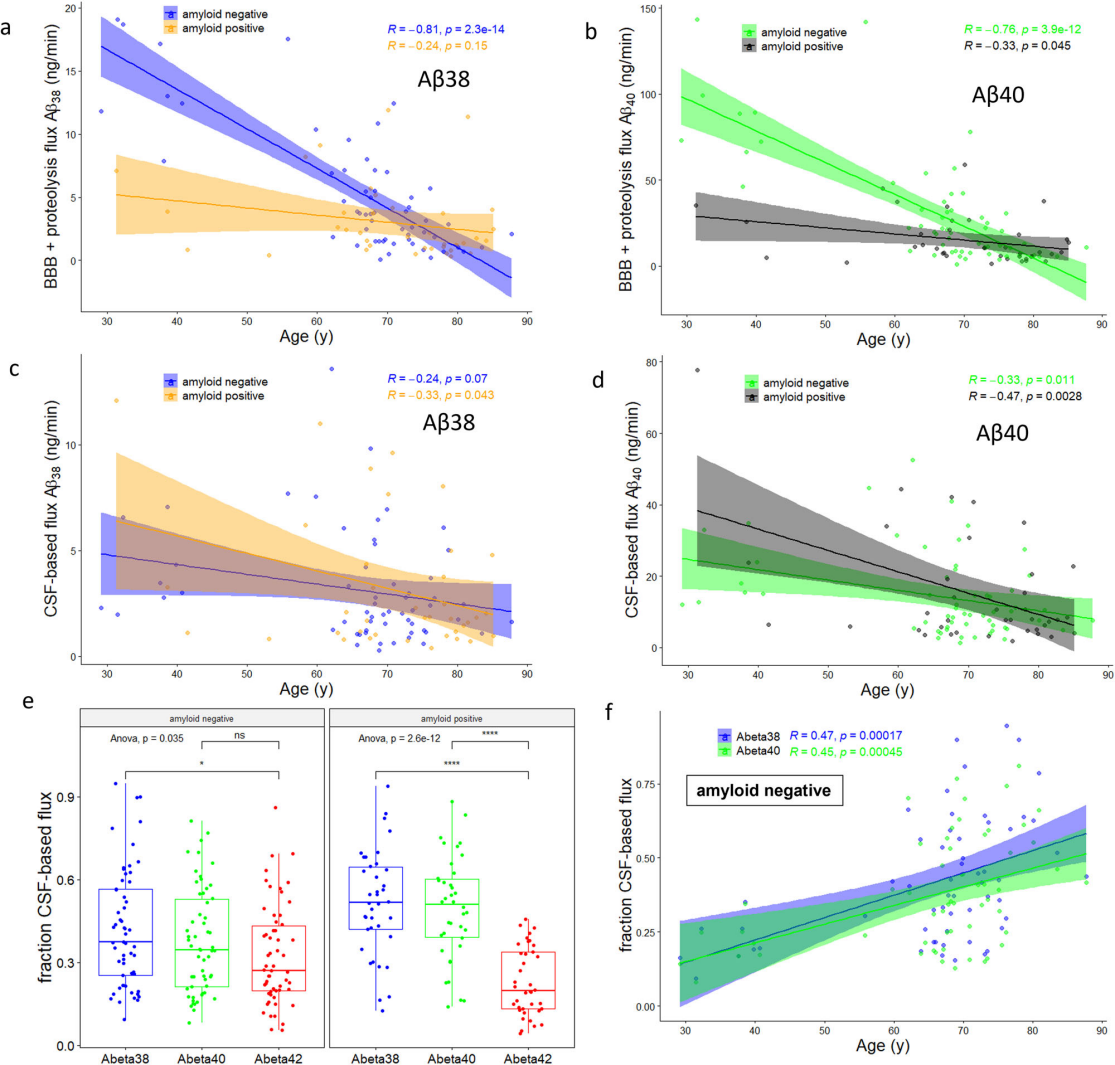
Derived Aβ mass fluxes. Importance of CSF-based clearance increases with age largely due to changes in clearance within the ISF. Clearance of: **a** Aβ_38_ and **b** Aβ_40_ from brain ISF declined with age in amyloid-negative subjects, but not in amyloid-positive subjects. In contrast, clearance of: **c** Aβ_38_ and **d** Aβ_40_ from CSF (and thus also by transfer from ISF to CSF) was weakly correlated with age. **e** The fraction of Aβ_42_ cleared by CSF-based processes was significantly lower than that of Aβ_38_ and Aβ_40_ in amyloid-positive subjects. This may be due to increased Aβ_42_ deposition into plaques in amyloid-positive subjects (Table 1), which may lower the brain ISF/cranial CSF concentration gradient that contributes to ISF-to-CSF clearance. *P < 0.05, ****P < 0.0001. **f** The fraction of CSF-based clearance significantly increased with age for Aβ_38_ and Aβ_40_ in amyloid-negative subjects due to the significant decline in clearance across the blood–brain barrier or by proteolysis (also see Table 1).

The model predicts that the fluxes of Aβ peptides across the BBB and via proteolysis decrease dramatically in normal aging (Fig. 5a, b). However, the presence of amyloid plaques depresses BBB and proteolysis fluxes (Table 1) and mutes the age effect (Fig. 5a, b). In stark contrast, the CSF-based flux and thus the ISF-to-CSF flux was only weakly correlated with age (Fig. 5c, d). However, the CSF-based and ISF-to-CSF flux became more important with age, mostly because of the dramatic decline in BBB and proteolysis fluxes (Fig. 5e). By amyloid status, the fraction of Aβ clearance that was due to ISF-to-CSF transfer increased for Aβ_38_ and Aβ_40_ but decreased for Aβ_42_ (Table 1). The decrease of Aβ_42_ in amyloid-positive subjects (Fig. 5f) is likely related to the large deposition flux of Aβ_42_ in the presence of amyloid plaques (Table 1). The magnitudes of the pathways also differed in amyloid-negative subjects. CSF-based flux of Aβ_38_ was 3.1 (0.3) ng/h, while BBB + proteolysis flux was 5.5 (0.7) ng/h (*n* = 58; *P* = 0.003). CSF-based flux of Aβ_40_ was 14 (2) ng/h, while BBB + proteolysis flux was 31 (4) ng/h (*n* = 58; *P* = 0.0005). CSF-based flux of Aβ_42_ was 2.2 (0.2) ng/h, while BBB + proteolysis flux was 4.5 (0.6) ng/h (*n* = 58; *P* = 0.0006).

**Fig. 6:**
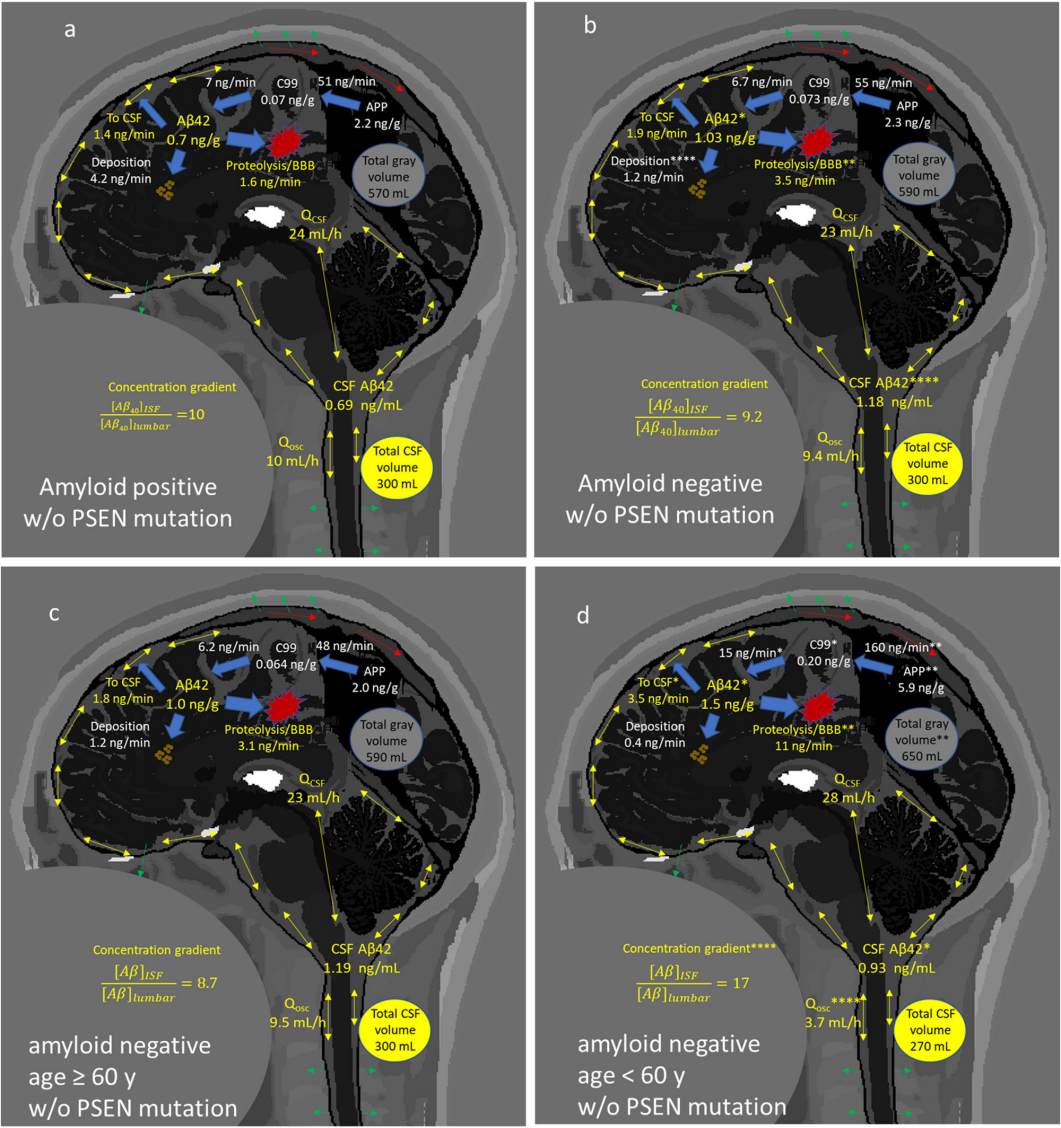
Graphical summary of major results by amyloid status and age. Summary of major findings. **a**, **b** In non-mutation carriers, the presence of amyloid plaques decreases the CSF and ISF concentration of Aβ_42_, due to enhanced deposition. This is despite the decreased mass flux across the blood–brain barrier or due to proteolysis, and a decrease in transfer from ISF to CSF. *t* test of predicted marginal means at age = 69.9 y: *P < 0.05, **P < 0.01, ***P < 0.001, ****P < 0.0001. **c**, **d** In older amyloid-negative, non-mutation carriers, total gray volume and production of APP are reduced relative to younger subjects. This decreases the flux toward Aβ peptides, including Aβ_42_. However, the ISF concentration of Aβ_42_ is only slightly elevated in younger subjects, because of greater fluxes across the BBB or due to proteolysis and greater ISF-to-CSF transfer. In younger subjects, the concentration gradient of Aβ_42_ from the ISF to lumbar space is larger, due to a smaller *Q*_*osc*_. *t* test: *P < 0.05, **P < 0.01, ***P < 0.001, ****P < 0.0001. See Supplementary Table 6 for complete results.

The major results of the study are summarized in Fig. 6, with full results presented in Supplementary Table 6. The presence of amyloid-β plaques is associated with a decrease in the ISF and CSF concentrations of Aβ_42_, a decrease in the BBB/proteolysis fluxes of Aβ_42_, and an increase in deposition flux of Aβ_42_ (Fig. 6a, b). In normal aging (Fig. 6c, d), a decrease in the cortical concentration of Aβ is predicted by the model, which has been previously observed^85^. The ISF-to-lumbar CSF concentration gradient is also predicted to be greater in younger subjects, associated with a lower *Q*_*osc*_. Much greater clearance of Aβ_42_ via BBB/proteolysis is found in younger subjects, with a smaller increase in CSF-based clearance. These balance the greater rate of production of APP- > C99- > Aβ_42_, perhaps due to greater neuron number reflected in the larger volume of gray matter or due to greater neuronal activity.

### Conclusion

The age-related decline in Aβ peptide turnover has now been ascribed largely to changes in proteolysis and/or blood–brain barrier transport. While the current model and dataset cannot distinguish proteolysis from BBB transport, both should receive enhanced focus as targets for early AD interventions.

## Methods

### Human studies

The human studies were performed at Washington University in St. Louis. The studies were approved by the Washington University Human Studies Committee and the General Clinical Research Center Advisory Committee. Participants completed informed written consent. The current results are from a subset of 100 subjects who completed the SILK study and MRI imaging scans. Subject demographics are summarized in Supplementary Table 1.

### SILK, MRI, and PET

SILK data collection procedures and PET PIB measurements were previously described^1,2^. Briefly, subjects received intravenous and intrathecal lumbar catheters between 7 a.m. and 9 a.m. The collection of samples began between 8 a.m. and 10 a.m. Time zero CSF and plasma baseline samples were withdrawn, then 3 mg/kg l-[U-^13^C_6_]leucine was given as a bolus for 10 min, followed by a constant infusion of 2 mg/kg/h for the remainder of the first 9 h. Blood samples (12 mL) were obtained hourly for the first 16 h and every other hour for the rest of the experiment (36 h). CSF (6 mL) was withdrawn hourly. The deposition of amyloid plaques was quantified by [^11^C]PIB-PET in 77 subjects. Subjects with mean cortical binding potential (MCBP) score >0.18 were considered to be amyloid positive. If PET PIB was not available, a lumbar CSF concentration ratio Aβ_42_/Aβ_40_ < 0.12 was considered to be amyloid positive. PET PIB scans were performed within 3 years before or after the SILK tracer study date. MRI scans (3 T volumetric T1-weighted) were processed using FreeSurfer version 5.3 as previously described^86^. FDA, Center for Devices and Radiological Health, and IT’IS Foundation collected the MRI data used to generate the images in Fig. 6^87^.

### Mass spectrometry

Sample processing and mass spectrometric measurements were previously described^1,2^. Briefly, Aβ peptides were immunoprecipitated with a mid-domain antibody, in the presence of isotope-labeled internal standard peptides. Concentrations of peptides and fractional isotope labeling were quantified by LC-MS/MS analysis on a Xevo TQ-S (Waters) or TSQ Vantage (ThermoScientific) mass spectrometer. Isotope-labeled leucine was captured from the blood by cation exchange chromatography. The N-heptafluorobutyryl n-propyl ester derivative was quantified by GCMS (Agilent 6890N GC and Agilent 5973N Mass Selective Detector).

### Mathematical model

Model equations were solved numerically in Julia as described in Supplementary Methods 1. Parameter meaning, values, and sources are shown in Supplementary Table 7.

The parameters that were fit to the model were: *k*_*BPD38*_, *k*_*BPD40*_*, k*_*BPD42*_*, SF*_*38*_*, SF*_*40*_*, SF*_*42*_*, Q*_*osc*_*, Q*_*CSF*_*, k*_*ex42*_. Other parameters were estimated as follows:

1. The volumetric rate of CSF production was *Q*_*CSF*_, which was assumed to arise solely from the choroid plexus. Extravasation of fluid from the capillaries of the brain parenchyma was not considered in the model. It was assumed that the concentration of Aβ peptides in newly formed CSF was zero.
2. *V*_*ISF*_ represents the volume of brain gray matter ISF, which was 10% of MRI-derived total gray volume. *V*_*cranial*_ is the volume of the cranial SAS. *V*_*CV*_ is the volume of the cisterns and ventricles, which are assumed to be in intimate communication due to oscillatory flow. Nominally, the cervical spinal volume is *V*_*SP1*_, the thoracic spinal volume is *V*_*SP2*,_ and the lumbar spinal volume is *V*_*SP3*_.
3. CSF was withdrawn from the lumbar catheter at the start of each hour and the model assumptions are summarized in Fig. 1b–e. The volume of CSF removed (6 mL) was divided by the time required for withdrawal (assumed to be 6 min for all subjects, actual range was stated by the practitioners to be 5–10 min). This yielded the volumetric flow rate of *V*_*LP*_*/t*_*CSF draw*_ *=* 60 mL/h, where *V*_*LP*_ is 6 mL and *t*_*CSF draw*_ is 0.1 h. It was assumed that the net caudal flow could not exceed *Q*_*CSF*_ during CSF withdrawal. If *V*_*LP*_*/t*_*CSF draw*_ exceeded *Q*_*CSF*_, which it did for all subjects, it was assumed that the excess flow came exclusively from a decrease in volume of the lumbar space (*V*_*SP3*_), which has been observed in humans during lumbar puncture^51^. The parameter *Q*_*LP*_ represented net caudal flow during withdrawal and refill, while *Q*_*refill*_ was used to account for volumetric changes in the lumbar compartment.
4. Following CSF withdrawal, it was assumed that the entirety of *Q*_*CSF*_ was used to refill the lumbar volume to its natural dimensions. The time required to refill the lumbar space was Δ*V*_*SP3*_*/Q*_*CSF*_.
5. After the lumbar space was fully refilled, *Q*_*LP*_ = 0 for the rest of the hour. However, if a CSF leak was suggested by the lumbar Aβ concentration data, then *Q*_*LP*_ = *Q*_*leak*_, where *Q*_*leak*_ is a constant during the entire 36–48 h SILK experiment, unless otherwise modified to better fit the concentration rise data. The maximum value for *Q*_*leak*_ was assumed to be *Q*_*CSF*_. During CSF withdrawal and lumbar space refilling, it was assumed that the drop in pressure halted the leak, i.e.*, Q*_*leak*_ = 0 during withdrawal and refilling.
6. Some concentration-time courses suggested step changes in *Q*_*leak*_ during the study. This might have occurred if the catheter shifted due to a change in the subject position. In these cases, an initial constant value of *Q*_*leak*_ was changed to another constant value at a certain time to better fit the lumbar Aβ concentration data.
7. The loss of CSF down spinal nerves, *Q*_*SN*_, was modeled as a net flow of CSF out of each spinal SAS compartment. It was assumed that *Q*_*SN*_ was divided equally between cervical, thoracic and lumbar compartments. The full flow entered the cervical space, but 1/3 *Q*_*SN*_ left down spinal nerves within the cervical space, while 2/3 *Q*_*SN*_ continued into the thoracic compartment. In the thoracic space, 1/3 *Q*_*SN*_ left down spinal nerves within the thoracic space, while 1/3 *Q*_*SN*_ continued into the lumbar space. The final 1/3 *Q*_*SN*_ left down spinal nerves in the lumbar space. During removal of CSF and refilling of the lumbar space, it was assumed that the drop in pressure completely halted loss of CSF down spinal nerves (*Q*_*SN*_ = 0). The model-predicted cisternography half-life was sensitive to *Q*_*SN*_. *Q*_*SN*_ was varied between 5 and 20% of *Q*_*CSF*_. The Moriyama et al. model predicted a mean half-life for all subjects of 20 ± 2.4 h^67^. The mean cisternography half-life predicted by the current model best agreed with the Moriyama et al. prediction when *Q*_*SN*_ equaled 10% of *Q*_*CSF*_ (Supplementary Table 8). In a study in sheep, the average of three methods indicated that 20.6% of CSF was absorbed by spinal nerves^69^. Other studies have indicated higher values, e.g., 38% in resting humans^54^.
8. The loss of CSF from the cranial space (e.g., at arachnoid granulations, dural lymphatics, cribriform plate, etc.) was *Q*_*cranial*_ = *Q*_*CSF*_ − *Q*_*SN*_ *− Q*_*LP*_. This reflected that the outflow from the cranial space was only a part of the total CSF produced by the choroid plexus. During CSF withdrawal and refilling, *Q*_*SN*_ = 0 and *Q*_*LP*_ = *Q*_*CSF*_, so *Q*_*cranial*_ = 0.
9. *Q*_*osc*_ and *Q*_*glymph*_ represent the oscillatory CSF flow generated by the cardiac and respiratory cycles but are modeled as steady bidirectional flows instead of pulsatile flows due to computational and MRI data constraints. In both cases, mass transport would only occur in the presence of a concentration difference between compartments.
10. The exchange compartment was only relevant to Aβ_42_ and was implemented as in the previous steady-state model^3^.
11. The SILK isotope-labeling data were also scaled by three additional free parameters, the scaling factors (SF_38_, SF_40_, and SF_42_). These are believed to account for instrument calibration errors and were previously described^1–3^.
12. The Michaelis–Menten rate constants for conversion of APP to C99 + C83 and C99 to Aβ_38–42_ were determined by Ortega et al^56^. Because the rate constants were given in relative units, units for the rate constants were derived by comparison with the carefully performed study of APP conversion to C99 (see Supplementary Table 9)^57^. The production rate of APP (*k*_*f*_), *V*_*max,gamma38*_ and *V*_*max,gamma42*_ were determined from solution of the steady-state equations. An example of labeling kinetics from plasma leucine to the lumbar SAS is shown in Supplementary Fig. 4.
13. APP is a transmembrane protein largely expressed by neurons in the brain. It appears to react with beta-secretase on the cell exterior and gamma-secretase within endosomes^79^, which are both topologically extracellular. Because the Michaelis–Menten rate constants were determined in cultured cells in a relative manner^56^, and for beta-secretase kinetics in an absolute manner in human temporal cortex preparations^57^, it was assumed that all of the rate constant values were applicable to the neuronal plasma membrane found in cortical tissue. The volume of ISF was assumed to be 10% of the total gray volume^80^, and the total gray volume was determined for each subject by MRI (Supplementary Fig. 5). Aβ peptides were assumed to be directly released into the ISF and all other species were transmembrane spanning. The intracellular concentration of Aβ peptides was assumed to be zero and the volume of endosome/lysosomes to be negligible.
14. The parameter *Q*_*glymph*_ accounts for the exchange of Aβ between CSF and ISF but was not well defined by the SILK data when set as a free parameter. The concentration “gradient” [Aβ_40_]_ISF_/[Aβ_40_]_lumbar_ was sensitive to the glymphatic exchange flow rate parameter (*Q*_*glymph*_). In pilot studies, when *Q*_*glymph*_ was decreased from *Q*_*CSF*_ to *Q*_*CSF*_/2, the lumbar-to-ISF concentration rise increased from 9.6 ± 6.9 to 14 ± 11. Further decreasing *Q*_*glymph*_ by setting it equal to *Q*_*osc*_ (slightly less than Q_CSF_/2) resulted in a lumbar-to-ISF concentration rise of 71 ± 130. Given the similarity between Aβ concentrations measured in homogenized cortex and CSF, lower values of *Q*_*glymph*_ were ruled out and *Q*_*glymph*_ was set equal to *Q*_*CSF*_ for all subjects. The governing assumption was that soluble Aβ exists entirely in ISF and that homogenization of cortical tissue dilutes the ISF about tenfold with intracellular fluids, thus decreasing the measured concentration by a factor of 10. With this assumption, cortical ISF concentrations are predicted to be tenfold higher than those measured in the homogenized cortex. In the final model with *Q*_*glymph*_ = *Q*_*CSF*_, the mean lumbar-to-ISF concentration rise was 10 ± 6.6.
15. For each equation shown (except for dV_SP3_/dt), isotopically labeled and unlabeled peptides were modeled separately. The APP production rate (*k*_*f*_) was scaled by *f*_*Leu*_ (the fraction of labeled leucine) for production of labeled APP, and scaled by (1 − *f*_*Leu*_) for production of unlabeled APP. It was assumed that the fraction of labeled leucine in ISF was the same as the fraction of labeled leucine in plasma, which was measured hourly for the first 13 h, then at 17 and 35 h. The tail of the plasma leucine labeling curve was fit to an exponential model^2^.

### Statistics and reproducibility

Statistical analyses were performed in R and comparisons were by two-sided *t* test with unequal variances or ANOVA with Tukey post hoc analysis. Errors are standard deviations except where noted. Box plots show: center line = median; box limits = upper and lower quartiles; whiskers = 1.5× interquartile range; points = outliers. Confidence interval region = 95%. Measurements were taken from distinct samples. Replicates are biological replicates.

### Reporting summary

Further information on research design is available in the Nature Research Reporting Summary linked to this article.

## Supplementary information

Supplementary Materials

Description of Additional Supplementary Files

Supplementary Data 1

Supplementary Data 2

Supplementary Data 3

Reporting Summary

## Data Availability

De-identified source data (Supplementary Data 1) and model results (Supplementary Data 2 and 3) are included in the Supplementary Materials.

## Code availability

Code deposited at https://zenodo.org/badge/latestdoi/437570053.
