# Supplementary material for: Importance of CSF-based Aβ clearance with age in humans increases with declining efficacy of blood-brain barrier/proteolytic pathways
Source: Commun Biol. 2022 Jan 27;5:98. doi: 10.1038/s42003-022-03037-0 (PMC8795390; doi:10.1038/s42003-022-03037-0)
Supplement: Supplementary file 1 — Supplementary Materials [file 42003_2022_3037_MOESM1_ESM.pdf]

Importance of CSF-based Aβ clearance with age in humans increases with declining efficacy of blood-brain barrier/proteolytic pathways

Supplementary Materials

Submitted to: *Communications Biology*

Supplementary Table 1: Subject characteristics

		Amyloid negative (mean ± S.D.)	Amyloid positive (mean ± S.D.)	p-value[4]
Demographics	Total subjects (#)	58	38	
	Female (%)	51.7	47.4	
	Age (y)	65.8 ± 13.4	70.3 ± 12.7	0.10
	Height (in)	67.0 ± 4.18	66.0 ± 3.52	0.24
	Weight (lb)	179 ± 40.6	161 ± 29.7	***0.013***
	BMI	28.1 ± 5.82	25.9 ± 4.19	***0.036***
	PET-PIB performed (#)	51	26	
	ApoE4 carriers (#)	11	27	
	PSEN mutation (#)	4	5	
Study	LOAD (#)	44	33	
	FACS (#)	14	5	
Lumbar CSF	[Tau] (pg/mL)	291 ± 244[1]	510 ± 186[2]	***5.0 x 10^{-5}***
	[pTau] (pg/mL)	49.0 ± 22.5[1]	89.7 ± 45.5[2]	***5.6 x 10^{-5}***
	[Aβ42] (ng/mL)	1.2 ± 0.44	0.68 ± 0.16	***2.9 x 10^{-11}***
	[Aβ42]/ [Aβ40]	0.16 ± 0.034	0.094 ± 0.017	***1.2 x 10^{-22}***
Steady state model[3]	FTR38	0.095 ± 0.035	0.085 ± 0.026	0.13
	FTR40	0.10 ± 0.036	0.09 ± 0.025	***0.046***
	FTR42	0.11 ± 0.044	0.13 ± 0.054	***0.027***
	FTR42/FTR40	1.1 ± 0.21	1.5 ± 0.29	***4.7 x 10^{-10}***
	Production rate ratio ($k_{A\beta42}/k_{A\beta40}$)	0.17 ± 0.027	0.14 ± 0.031	***2.6 x 10^{-7}***
	k_{ex42} (h^{-1})	0.0031 ± 0.035	0.084 ± 0.086	***1.7 x 10^{-6}***

Notes:

(1) n = 42

(2) n = 30

(3) Steady state model previously published in Patterson, B. W. *et al.* Age and amyloid effects on human central nervous system amyloid-beta kinetics. *Ann. Neurol.* **78**, 439–453 (2015)

(4) t-test; *italics*: p < 0.05; ***bold***: p < 0.01

Supplementary Table 2 – Turnover parameters from the steady state model

Steady state model parameters	***Amyloid negative (n = 58)***		***Amyloid positive (n = 38)***	
	Correlation coefficient with age	***p-value***	***Correlation coefficient with age***	***p-value***
FTR38	*-0.79*	***1.2×10^{-13}***	-0.48	***2.1×10^{-3}***
FTR40	*-0.76*	***6.9×10^{-12}***	-0.43	***7.6×10^{-3}***
FTR42	*-0.60*	***7.5×10^{-7}***	-0.54	***5.1×10^{-4}***
FTR42/FTR40	*0.14*	0.30	-0.49	***1.6×10^{-3}***
Production rate ratio ($k_{A\beta42}/k_{A\beta40}$)	*0.34*	***8.9×10^{-3}***	-0.54	***5.1×10^{-4}***
k_{ex42} (h^{-1})	*-0.034*	0.80	-0.37	**0.020**

Steady state model previously published in Patterson, B. W. *et al.* Age and amyloid effects on human central nervous system amyloid-beta kinetics. *Ann. Neurol.* **78**, 439–453 (2015). t-test; *italics*: $p < 0.05$; ***bold***: $p < 0.01$

Supplementary Table 3 – MRI brain volumes, thicknesses and derived measures, differences by amyloid status (amyloid negative n = 58; amyloid positive n = 38)[1]

	Brain region	Amyloid negative	Amyloid positive	p-value
MRI volume (µL) differences with $p < 0.001$	Amygdala_vol	1550 ± 260	1220 ± 237	***8.0 x 10⁻⁹***
	Hippocampus_vol	3750 ± 515	3160 ± 559	***1.3 x 10⁻⁶***
	Precuneus_vol	9470 ± 1480	8380 ± 1070	***6.2 x 10⁻⁵***
	Supramarginal_vol	10,100 ± 1450	9040 ± 1230	***1.3 x 10⁻⁴***
	Inferiorparietal_vol	13,200 ± 2070	11,700 ± 1640	***2.3 x 10⁻⁴***
	Accumbensarea_vol	539 ± 133	446 ± 105	***2.5 x 10⁻⁴***
MRI cortical thickness (mm) differences with $p < 0.001$	inferiorparietal_thick	2.46 ± 0.122	2.33 ± 0.148	***4.4 x x 10⁻⁵***
	fusiform_thick	2.66 ± 0.153	2.55 ± 0.128	***1.6 x 10⁻⁴***
	precuneus_thick	2.32 ± 0.115	2.22 ± 0.153	***4.2 x 10⁻⁴***
	superiorparietal_thick	2.22 ± 0.114	2.11 ± 0.159	***8.0 x 10⁻⁴***
Summary MRI volumes (mL) differences with $p < 0.05$	TotalGrayVol	599 ± 68.1	566 ± 41.5	***0.0041***
	SupraTentorialVolNotVent	913 ± 114	866 ± 70.4	*0.015*
	Ventricle brain ratio (ventricle volume/total brain parenchyma volume)[2]	0.033 ± 0.017	0.043 ± 0.022	*0.020*
	VentricleVol	35.8 ± 18.0	45.9 ± 24.9	*0.035*
	Ventricle CSF vol (Total_CSF_vol)	32.5 ± 17.4	42.2 ± 24.3	*0.037*

[1] Derived using Freesurfer version 5.3. Where applicable, values for each subject were the average of both cerebral hemispheres.

[2] Measure described in: Ott, B. R. *et al.* Brain ventricular volume and cerebrospinal fluid biomarkers of Alzheimer's disease. *J. Alzheimer's Dis.* **20**, 647–657 (2010)

t-test; *italics*: $p < 0.05$; ***bold***: $p < 0.01$

Supplementary Table 4 – MRI brain volumes, thicknesses and derived measures, correlation with age in amyloid negative subjects (n = 58)

Brain region	***Correlation coefficient with age***	***p-value***
Putamen volume	*-0.70*	1.3×10^{-9}
Accumbens-area volume	*-0.67*	8.9×10^{-9}
Thalamus Proper volume	*-0.60*	6.8×10^{-7}
Hippocampus volume	*-0.59*	1.4×10^{-6}
Banks of Superior Temporal Sulcus thickness	*-0.57*	2.5×10^{-6}
Lingual thickness	*-0.55*	9.5×10^{-6}
Ventricle brain ratio (ventricle volume/total brain parenchyma volume) [1]	*0.54*	1.1×10^{-5}
Superior temporal volume	*-0.51*	4.7×10^{-5}
Parstriangularis volume	*-0.50*	6.3×10^{-5}

[1] Measure described in: Ott, B. R. *et al.* Brain ventricular volume and cerebrospinal fluid biomarkers of Alzheimer's disease. *J. Alzheimer's Dis.* **20**, 647–657 (2010)

Supplementary Table 5 – Interactions between age and amyloid status

		Generalized linear model p-values (ANOVA)		
		Amyloid status	Age	Amyloid status x age
CSF leak	Q_{leak}	0.20	***9.6 x 10^-4^***	0.12
Aβ clearance	k_{BPD38} (h^{-1})	*0.033*	***7.0 x 10^-9^***	***2.6 x 10^-4^***
	k_{BPD40} (h^{-1})	***0.0050***	***1.0 x 10^-6^***	***8.5 x 10^-4^***
	k_{BPD42} (h^{-1})	***2.4 x 10^-5^***	***0.0020***	0.27
	k_{BPD42}/k_{BPD38}	***2.7 x 10^-6^***	0.64	0.19
	k_{BPD42}/k_{BPD40}	***1.6 x 10^-8^***	0.35	*0.019*
	k_{BPD40}/k_{BPD38}	0.088	*0.042*	0.66
Aβ production (w/o mutation carriers)	$V_{max,\gamma42}/ V_{max,\gamma38}$	0.73	0.35	***0.006***
	$V_{max,\gamma42}$ (μg/(mL·h))	0.60	0.13	***0.007***
APP production (w/o mutation carriers)	k_f (ng/h)	0.96	***3.1 x 10^-7^***	0.53
	Total Gray Volume (mL)	0.20	***2.6 x 10^-4^***	0.51
Exchange	k_{ex42} (h^{-1})	***3.6 x 10^-4^***	0.23	*0.024*
CSF Fluid flow	$Q_{CSF} = Q_{glymph}$ (mL/h)	0.40	0.088	*0.032*
	Q_{osc} (mL/h)	0.20	***9.6 x 10^-4^***	0.12
	$[A\beta_{40}]_{ISF}/[A\beta_{40}]_{lumbar}$	0.91	***6.4 x 10^-4^***	0.33
	Predicted cisternography half-life (h)	0.41	0.24	*0.025*
	V_{CSF} (mL/h)	0.55	***0.005***	0.17
Flux $A\beta_{38}$	*Glymphatic* (ng/min)	0.58	***0.007***	0.43
	BBB +proteolysis (ng/min)	*0.025*	***1.6 x 10^-14^***	***1.3 x 10^-6^***
	% glymphatic flux	0.058	***0.002***	*0.019*
Flux $A\beta_{40}$	*Glymphatic* (ng/min)	0.35	***8.9 x 10^-5^***	0.13
	BBB + proteolysis (ng/min)	*0.011*	***4.5 x 10^-14^***	***3.6 x 10^-6^***
	% glymphatic flux	***0.007***	*0.012*	*0.013*
Flux $A\beta_{42}$	*Glymphatic* (ng/min)	0.16	***1.5 x 10^-4^***	0.18
	BBB + proteolysis (ng/min)	***1.2 x 10^-4^***	***1.2 x 10^-11^***	***2.7 x 10^-5^***
	Deposition (ng/min)	***9.0 x 10^-6^***	***3.5 x 10^-4^***	***0.0080***
	% glymphatic flux	***0.001***	***0.005***	0.32

Supplementary Table 6: Summary of model results by amyloid status and age presented in Figure 6

		PSEN mutation negative Predicted marginal means (S.E.) at age = 69.9 y			Amyloid negative, PSEN mutation negative Mean (S.E.)		
		Amyloid positive	Amyloid negative	p-value	Age ≥ 60 N = 47	Age < 60 N = 7	p-value
Flow rate (mL/h)	Q_{CSF}	24 (2)	23 (1)	0.85	23 (1)	28 (4)	0.20
	Q_{osc}	10 (1)	9.4 (0.8)	0.69	9.5 (0.9)	3.7 (0.4)	***<0.0001***
Volume (mL)	V_{CSF}	300 (20)	300 (10)	0.82	300 (10)	270 (20)	0.10
	Total Gray Volume	570 (10)	590 (8)	0.15	590 (10)	650 (20)	***0.0060***
Conc. Ratio	$[A\beta_{40}]_{ISF}/[A\beta_{40}]_{lumbar}$	10 (1)	9.2 (0.8)	0.82	8.7 (0.7)	17 (1)	***<0.0001***
Brain Concentration (ng/g)	*Conc. APP*	2.2 (0.3)	2.3 (0.2)	0.79	2.0 (0.2)	5.9 (0.9)	***0.0050***
	C99	0.07 (0.01)	0.073 (0.007)	0.62	0.064 (0.006)	0.20 (0.03)	***0.0052***
	$A\beta_{42}$	0.7 (0.1)	1.03 (0.08)	*0.012*	1.0 (0.1)	1.5 (0.2)	*0.033*
Lumbar CSF Concentration (ng/mL)	$A\beta_{42}$	0.69 (0.07)	1.18 (0.05)	***<0.0001***	1.19 (0.06)	0.93 (0.08)	*0.025*
Flux (ng/min)	APP→C99	51 (8)	55 (5)	0.63	48 (5)	160 (20)	***0.0019***
	C99→ $A\beta_{42}$	7 (1)	6.7 (0.7)	0.58	6.2 (0.5)	15 (2)	*0.011*
	Glymphatic/CSF-based	1.4 (0.3)	1.9 (0.2)	0.14	1.8 (0.2)	3.5 (0.6)	*0.045*
	Deposition	4.2 (0.5)	1.2 (0.4)	***<0.0001***	1.2 (0.3)	0.4 (0.3)	0.063
	BBB + proteolysis	1.6 (0.5)	3.5 (0.4)	***0.0033***	3.1 (0.4)	11 (2)	***0.0056***

italics: $p < 0.05$; ***bold***: $p < 0.01$

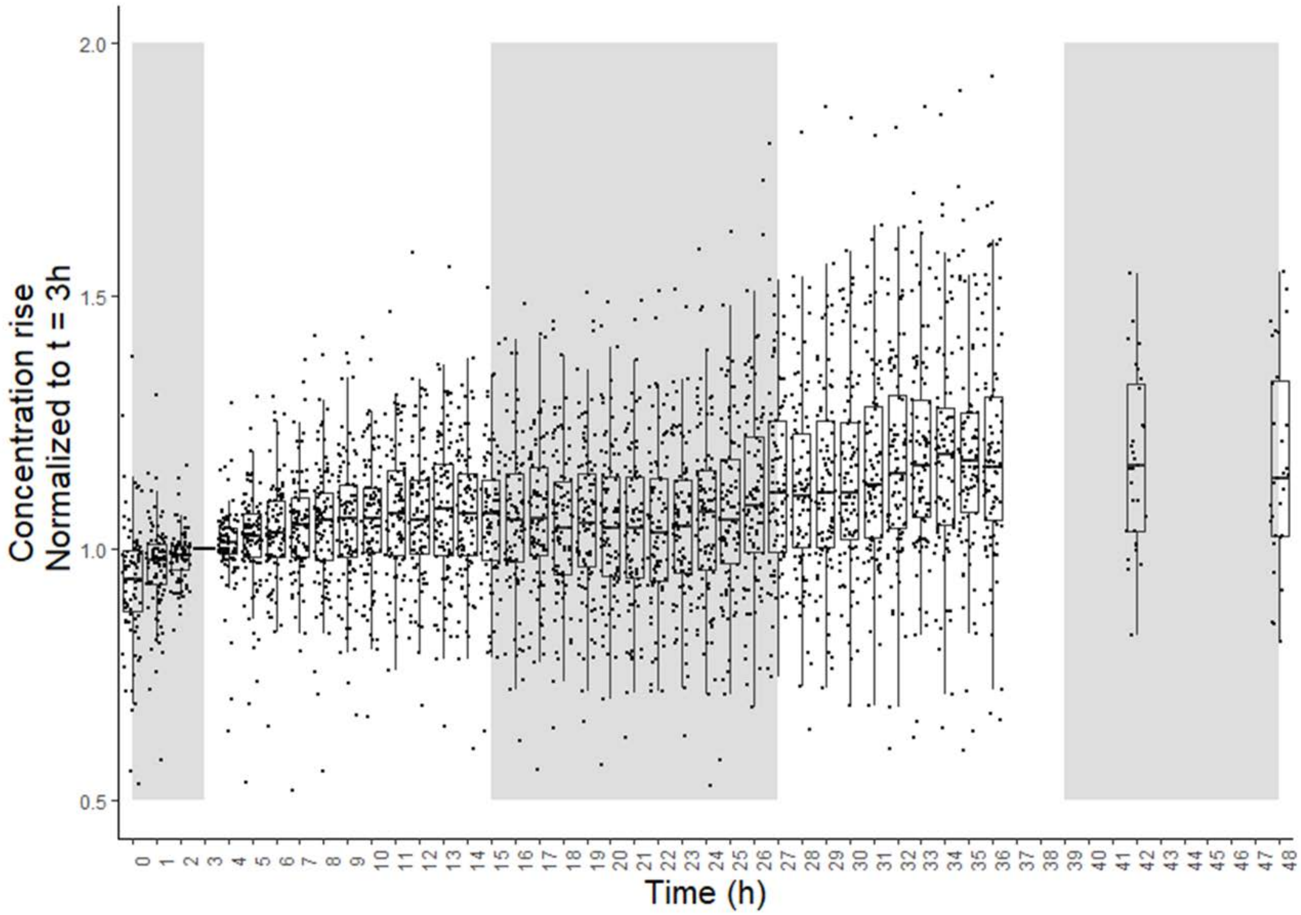

Supplementary Figure 1: Box plot of measured concentrations of Aβ peptides in the lumbar CSF during SILK experiment (n = 96).

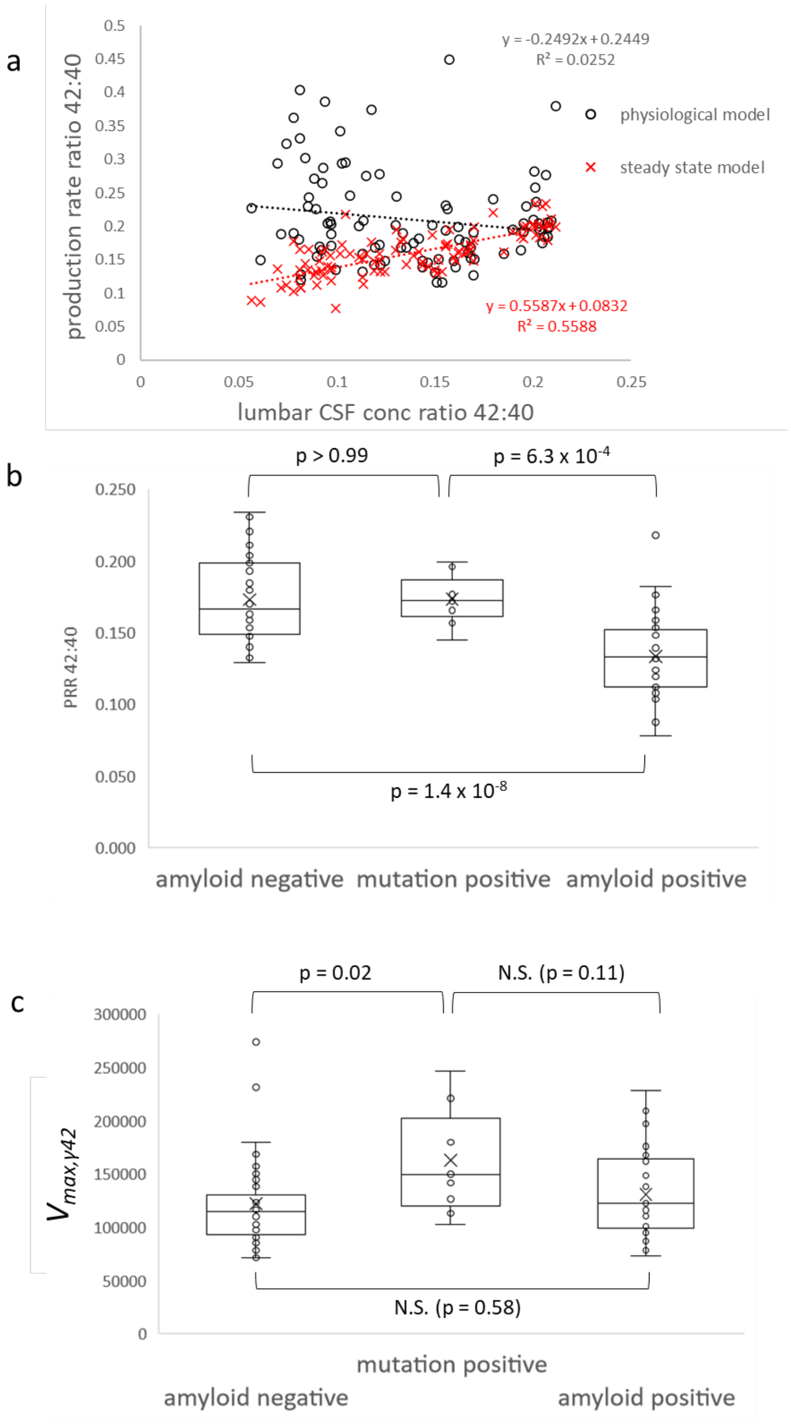

Supplementary Figure 2: a. In the previous steady state model, the production rate ratio was highly correlated with the lumbar CSF concentration ratio of $A\beta_{42}$:$A\beta_{40}$. The production rate in the steady state model is given exactly by:

$$Production\ rate\ ratio(A\beta_{42}: A\beta_{40}) = \frac{[A\beta_{42}]_{lumbar}}{[A\beta_{40}]_{lumbar}} \times \frac{FTR_{42}}{FTR_{40}}$$

This implied that the relative production of $A\beta_{42}$ declined with amyloidosis, for which no plausible mechanism exists. This implied that the ratio FTR42:FTR40 was underestimated in the presence of amyloid plaques. The current physiological model does not show a relationship between production rate ratio and lumbar concentration ratio. Steady state model previously published in Patterson, B. W. *et al.* Age and amyloid effects on human central nervous system amyloid-beta kinetics. *Ann. Neurol.* **78**, 439–453 (2015)

b. The production rate ratio (PRR42:40) from the steady state model was not higher in mutation carriers than in amyloid negative subjects. However, both were higher than amyloid positives. This is unexpected and related to the underestimation of FTR42:FTR40 as described above.

c. The V_{max} of gamma secretase for the production of $A\beta_{42}$ in the physiological model was significantly elevated in mutation carriers compared to amyloid negative subjects, but not compared to amyloid positive subjects. In previous studies, production rates for $A\beta_{42}$ were normalized by production rates for $A\beta_{40}$, which greatly reduced the coefficient of variation. However, gamma secretase V_{max} for $A\beta_{40}$ production was set to a fixed literature value for all subjects. Thus, V_{max} for $A\beta_{42}$ production was normalized by the V_{max} for $A\beta_{38}$ production in Figure 5d.

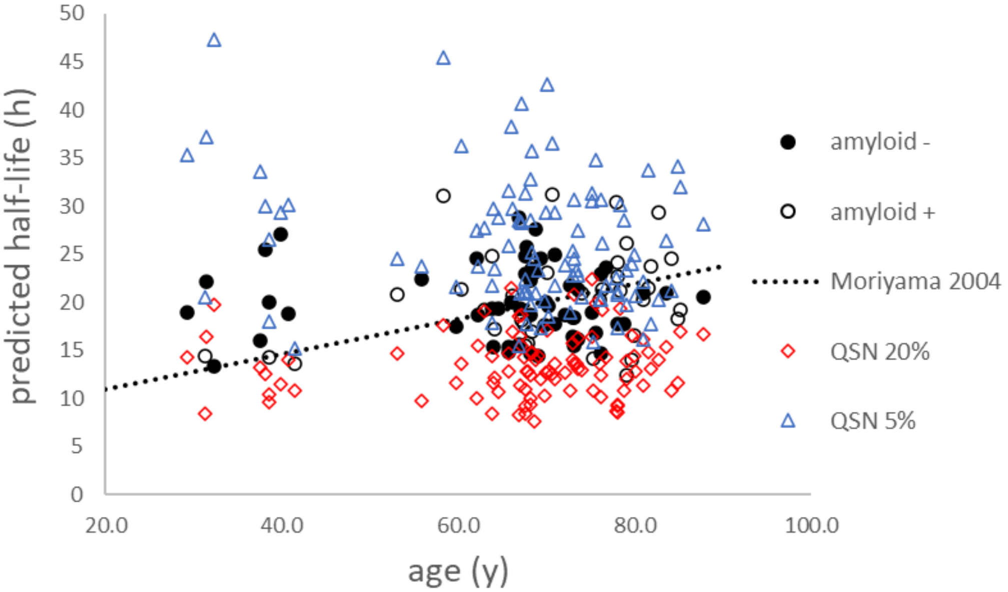

Supplementary Figure 3: The predicted cisternography half-life from the Moriyama model[1] and values predicted by the current physiological model. While the age-dependence noted with the physiological model was in the opposite direction (declining with age in amyloid negative subjects, r = -0.30, n = 58, p = 0.021), the mean cisternography half-life was well-matched when the percentage of CSF lost down spinal nerves (Q_{SN}) was 10%. The mean cisternography half-life was much shorter with Q_{SN} = 20% and much longer with Q_{SN} = 5%. Moriyama model published in: Moriyama, E., Ogawa, T., Nishida, A., Ishikawa, S. & Beck, H. Quantitative analysis of radioisotope cisternography in the diagnosis of intracranial hypotension. *J. Neurosurg.* **101**, 421–426 (2004)

Supplementary Methods 1: Model description

Model equations

The following system of differential equations was solved numerically, where $A\beta_x$ represents $A\beta_{38}$, $A\beta_{40}$ or $A\beta_{42}$. V_{gamma} and k_{BPD} were specific for each peptide. The exchange process was only active for $A\beta_{42}$.

$$\frac{d[APP]}{dt} = k_f - \frac{V_{M1}/K_{M1}\,[APP]}{1+[APP]/K_{M1}+[C99]/K_{M5}} - \frac{V_{M2}/K_{M2}\,[APP]}{1+[APP]/K_{M2}}$$

$$\frac{d[C99]}{dt} = \frac{V_{M2}/K_{M2}\,[APP]}{1+[APP]/K_{M2}} - \frac{\left(V_{gamma38}+V_{gamma40}+V_{gamma42}\right)/K_{M4}\,[C99]}{1+[C83]/K_{M3}+[C99]/K_{M4}} - \frac{V_{M5}/K_{M5}\,[C99]}{1+[APP]/K_{M1}+[C99]/K_{M5}}$$

$$\frac{d[C83]}{dt} = \frac{V_{M1}/K_{M1}\,[APP]}{1+[APP]/K_{M1}+[C99]/K_{M5}} + \frac{V_{M5}/K_{M5}\,[C99]}{1+[APP]/K_{M1}+[C99]/K_{M5}} - \frac{V_{M3}/K_{M3}\,[C83]}{1+[C83]/K_{M3}+[C99]/K_{M4}}$$

$$\begin{aligned}\frac{d[A\beta x]_{ISF}}{dt} = {} & \frac{V_{gamma}/K_{M4}\,[C99]}{1+[C83]/K_{M3}+[C99]/K_{M4}} - (k_{BPD}+k_{ex})[A\beta x]_{ISF} + k_{ret}[A\beta x]_{ex} \\ & + \frac{Q_{glymph}}{V_{ISF}}\left([A\beta x]_{cranial} - [A\beta x]_{ISF}\right)\end{aligned}$$

$$\frac{d[A\beta x]_{cranial}}{dt} = \frac{Q_{glymph}}{V_{cranial}}\left([A\beta x]_{ISF} - [A\beta x]_{cranial}\right) + \frac{(Q_{cranial}+Q_{osc})}{V_{cranial}}\left([A\beta x]_{CV} - [A\beta x]_{cranial}\right)$$

$$\frac{d[A\beta x]_{CV}}{dt} = \frac{Q_{CSF}}{V_{CV}}\left([A\beta x]_{plasma} - [A\beta x]_{CV}\right) + \frac{Q_{osc}}{V_{CV}}\left([A\beta x]_{cranial} - 2[A\beta x]_{CV} + [A\beta x]_{SP1}\right)$$

$$\frac{d[A\beta x]_{SP1}}{dt} = \frac{Q_{osc}}{V_{SP1}}\left([A\beta x]_{CV} - 2[A\beta x]_{SP1} + [A\beta x]_{SP2}\right) + \frac{(Q_{LP}+Q_{SN})}{V_{SP1}}\left([A\beta x]_{CV} - [A\beta x]_{SP1}\right)$$

$$\frac{d[A\beta x]_{SP2}}{dt} = \frac{Q_{osc}}{V_{SP2}}\left([A\beta x]_{SP1} - 2[A\beta x]_{SP2} + [A\beta x]_{SP3}\right) + \frac{\left(Q_{LP}+\frac{2}{3}Q_{SN}\right)}{V_{SP2}}\left([A\beta x]_{SP1} - [A\beta x]_{SP2}\right)$$

$$\frac{dmass_{A\beta x,SP3}}{dt} = Q_{osc}\left([A\beta x]_{SP2} - [A\beta x]_{SP3}\right) + \left(Q_{LP}+\tfrac{1}{3}Q_{SN}\right)\left([A\beta x]_{SP2} - [A\beta x]_{SP3}\right) + Q_{refill}[A\beta x]_{SP3}$$

$$\begin{aligned}\frac{d[A\beta x]_{SP3}}{dt} &= \frac{d\left(mass_{A\beta x,SP3}/V_{sp3}\right)}{dt} = \frac{1}{V_{sp3}}\frac{dmass_{A\beta x,SP3}}{dt} + mass_{A\beta x,SP3}\frac{d\left(1/V_{sp3}\right)}{dt} \\ &= \frac{1}{V_{sp3}}\frac{dmass_{A\beta x,SP3}}{dt} - \frac{mass_{A\beta x,SP3}}{{V_{sp3}}^2}\frac{dV_{sp3}}{dt}\end{aligned}$$

$$\frac{d[A\beta 42]_{ex}}{dt} = k_{ex}[A\beta 42]_{ISF} - k_{ret}[A\beta 42]_{ex}$$

$$\frac{dV_{sp3}}{dt} = Q_{refill}$$

$$Q_{refill} = \begin{cases} Q_{CSF} - \dfrac{V_{LP}}{t_{CSF\,draw}} & 0 < t - \lfloor t \rfloor < t_{CSF\,draw} \\ Q_{CSF} & t_{CSF\,draw} < t - \lfloor t \rfloor < \dfrac{V_{LP}}{Q_{CSF}} \\ 0 & \dfrac{V_{LP}}{Q_{CSF}} < t - \lfloor t \rfloor < 1 \end{cases}$$

$$Q_{LP} = \begin{cases} Q_{CSF} & 0 < t - \lfloor t \rfloor < t_{CSF\ draw} \\ Q_{CSF} & t_{CSF\ draw} < t - \lfloor t \rfloor < \frac{V_{LP}}{Q_{CSF}} \\ 0\ or\ Q_{leak} & \frac{V_{LP}}{Q_{CSF}} < t - \lfloor t \rfloor < 1 \end{cases}$$

$$Q_{SN} = \begin{cases} 0 & 0 < t - \lfloor t \rfloor < t_{CSF\ draw} \\ 0 & t_{CSF\ draw} < t - \lfloor t \rfloor < \frac{V_{LP}}{Q_{CSF}} \\ Q_{SN} & \frac{V_{LP}}{Q_{CSF}} < t - \lfloor t \rfloor < 1 \end{cases}$$

ISF = brain interstitial fluid

CV = cisterns and ventricles

SP = spinal SAS

ex = exchange

ret = return

LP = lumbar puncture

SN = spinal nerve

osc = oscillatory

glymph = glymphatic

gamma = gamma secretase

Model parameters

Supplementary Table 7: Parameter meaning, values and sources

Parameter	Interpretation	Value	Units	Source
$k_{BPD38,40,42}$	Transport across BBB, proteolysis, and deposition	Fit to SILK and lumbar concentration timecourses	1/h	
$SF_{38,40,42}$	Scaling factors for SILK data, needed due to non-linearities in mass spectrometer response as a function of sample amount		N/A	
Q_{osc}	Bi-directional flow rate between SAS compartments		mL/h	
Q_{CSF}	CSF Production rate		mL/h	
k_{ex42}	Exchange of $A\beta_{42}$ with presumably existing amyloid plaques		1/h	
V_{ISF}	Volume of ISF	10% of TotalGrayVol	mL	Bender, B. & Kiose, U. Cerebrospinal fluid and interstitial fluid volume measurements in the human brain at 3T with EPI. Magn. Reson. Med. 61, 834–841 (2009)
TotalGrayVol	Total gray matter volume from FreeSurfer analysis of MRI scans	"the sum of lhCortex + rhCortex + SubCortGray + CerebellumGM"	µL	https://surfer.nmr.mgh.harvard.edu/fswiki/MorphometryStats
V_{CSF}	Volume of cranial, cisternal and ventricular CSF	Estimated from FreeSurfer 'EstimatedTotalIntraCranialVol' minus ('SupraTentorialVol', 'CerebellumCortex_vol', 'CerebellumWhiteMatter_vol', 'BrainStem_vol') and brain thickness fraction	mL	

		Allowed to vary to better fit lumbar concentration timecourse		
brain thickness fraction	Measure of distance from ventricle to brain surface	See description below	N/A	
$V_{cranial}$	Volume of CSF in cranial SAS	VCSF x 0.838	mL	Bottan, S., Poulikakos, D. & Kurtcuoglu, V. Phantom model of physiologic intracranial pressure and cerebrospinal fluid dynamics. IEEE Trans. Biomed. Eng. 59, 1532–1538 (2012)
V_{CV}	Volume of CSF in cisterns and ventricles	VCSF x 0.162 + FreeSurfer 'TotalCSF'	mL	
V_{SP1}, V_{SP2}, V_{SP3}	Volumes of cervical, thoracic and lumbar SAS	Literature values applied uniformly to all subjects, total volume of 80 mL, allowed to vary to better fit lumbar concentration timecourse	mL	Alperin, N., Bagci, A. M., Lee, S. H. & Lam, B. L. Automated quantitation of spinal CSF volume and measurement of craniospinal CSF redistribution following lumbar withdrawal in idiopathic intracranial hypertension. Am. J. Neuroradiol. 37, 1957–1963 (2016)<br>Chazen, J. L. *et al.* Automated segmentation of MR imaging to determine normative central nervous system cerebrospinal fluid volumes in healthy volunteers. *Clin. Imaging* **43**, 132–135 (2017)<br>Sass, L. R. et al. A 3D subject-specific model of the spinal subarachnoid space with anatomically realistic ventral and dorsal spinal cord nerve rootlets. Fluids Barriers CNS 14, 1–16 (2017)
V_{LP}	Volume of hourly lumbar puncture withdrawal	6 during withdrawal, otherwise 0	mL each hour	
$t_{CSF\ draw}$	Time for CSF withdrawal	0.1	h	Range: 5-10 min (authors: RJB and BPL)
Q_{LP}	Rate of CSF withdrawal	$V_{LP}/t_{CSF\ draw}$	mL/h	
Q_{refill}	Rate of change of lumbar SAS volume during CSF withdrawal	Q_{CSF} - Q_{LP}	mL/h	
Q_{leak}	Rate of CSF leakage due to catheter	Allowed to vary to better fit lumbar concentration timecourse	mL/h	
Q_{SN}	Rate of loss of CSF down spinal nerves, or CSF absorption in spinal SAS	Q_{CSF} *x 10%*	mL/h	Strongly affects prediction of cisternography half-life, adjusted from 5% - 20%, with 10% selected as value that provides most reasonable value for cisternography half-life<br>Moriyama, E., Ogawa, T., Nishida, A., Ishikawa, S. & Beck, H. Quantitative analysis of radioisotope cisternography in the diagnosis of intracranial hypotension. *J. Neurosurg.* **101**, 421–426 (2004)
$Q_{cranial}$	Rate of loss of CSF from the cranial space	$Q_{CSF} – Q_{SN} – Q_{LP}$		

Q_{glymph}	Bi-directional flow rate between cranial SAS and brain ISF	Q_{CSF}		Strongly affects concentration of Aβ peptides in brain ISF. Chosen to achieve 10-fold gradient between brain ISF and lumbar concentrations of Aβ peptides.<br>• Wang, J., Dickson, D. W., Trojanowski, J. Q. & Lee, V. M.-Y. The levels of soluble versus insoluble brain Abeta distinguish Alzheimer's disease from normal and pathologic aging. Exp. Neurol. 158, 328–337 (1999)<br>• Roberts, K. F. *et al.* Amyloid-β efflux from the central nervous system into the plasma. *Ann. Neurol.* **76**, 837–844 (2014)<br>• Freeman, S. H., Raju, S., Hyman, B. T., Frosch, M. P. & Irizarry, M. C. Plasma Aβ levels do not reflect brain Aβ levels. *J. Neuropathol. Exp. Neurol.* **66**, 264–271 (2007)<br>• Lue, L. *et al.* Soluble Amyloid Beta Peptide Concentration as a Predictor of Synaptic Change in Alzheimer's Disease. *Am. J. Pathol.* **155**, 853–862 (1999)
k_f	Rate of synthesis of APP	Determined from parameters, steady state equations and measured lumbar concentrations of $A\beta_{38}$, $A\beta_{40}$ and $A\beta_{42}$	ng/ mL h	
cAPP	Cortex concentration of APP		ng/g	
cC83	Cortex concentration of C83		ng/g	
cC99	Cortex concentration of C99		ng/g	
$V_{max,gamma38}$	Maximum rate of C99→Aβ38		ng/ mL h	
$V_{max,gamma42}$	Maximum rate of C99→Aβ42		ng/ mL h	
$V_{max,gamma40}$	Maximum rate of C99→Aβ40	609,798	ng/ mL h	Ortega, F., Stott, J., Visser, S. A. G. & Bendtsen, C. Interplay between α-, β-, and γ-secretases determines biphasic amyloid-β protein level in the presence of a γ-secretase inhibitor. *J. Biol. Chem.* **288**, 785–792 (2013)<br>Stockley, J. H., Ravid, R. & Neill, C. O. Altered β-secretase enzyme kinetics and levels of both BACE1 and BACE2 in the Alzheimer's disease brain. *FEBS Lett.* **580**, 6550–6560 (2006)
f_{Leu}	Fraction of plasma Leu that is isotope labeled	Timecourse measured by GC-MS from plasma samples	N/A	
$[A\beta x]_{plasma}$	Concentration of Aβ peptides in CSF generated at choroid plexus	Assumed to be zero due to the low concentration of Aβ peptides in plasma compared to CSF and the filtering properties of the choroid plexus. Would allow consideration of the effects of	0 ng/mL	

		a leaky choroid plexus if non-zero		

Supplementary Table 8 – Cisternography half-life predicted by current model

Q_{SN} (% of Q_{CSF})	*Predicted cisternography half-life (h)*
5	*27 ± 7.4*
10	*21 ± 4.5*
20	*14 ± 3.2*

Supplementary Table 9: Rate constants for enzymatic production of Aβ. ‘x’ notates the listed reaction.

	Secretase	x	K_{Mx} (ng/mL)	$V_{max,x}$ (ng/mL h)
APP → C83	α	1	2578.063	392,228.7
APP → C99	β	2	22,731.31	54,555.45
C83 → p3	γ	3	399,184	5,205,945
C99 → Aβ40	γ	4	12,682.41	609,737.72
C99 → C83	α	5	931.4293	7951.546

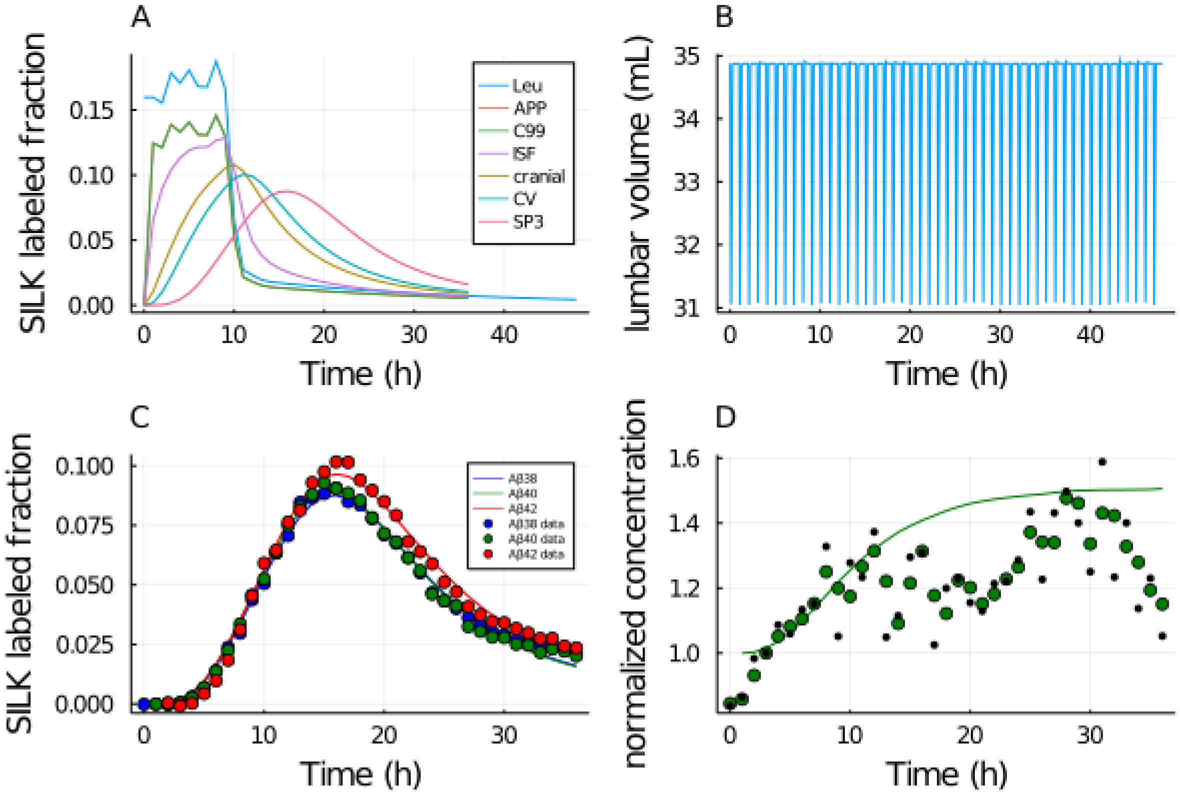

Supplementary Figure 4: *Example of results from simulation.* A. Timecourse for isotope labeling of proteins and peptides in a amyloid negative subject. Leu = plasma Leucine, APP = APP in neuronal membrane, C99 = C99 in neuronal plasma membrane, ISF = $A\beta_{38}$ in cortical interstitial fluid, cranial = $A\beta_{38}$ in cranial SAS, CV= $A\beta_{38}$ in cistern/ventricles, SP3 = $A\beta_{38}$ in third spinal compartment, *i.e.* lumbar SAS. B. Lumbar volume, response to hourly withdrawal of 6 mL. Because the rate of CSF withdrawal is large compared to the rate of CSF production, a volume decrease must occur in the system, which MRI studies suggest is mainly in the lumbar space (Alperin, N., Bagci, A. M., Lee, S. H. & Lam, B. L. Automated quantitation of spinal CSF volume and measurement of craniospinal CSF redistribution following lumbar withdrawal in idiopathic intracranial hypertension. Am. J. Neuroradiol. 37, 1957–1963 (2016)). C. Fits to SILK data. D. Fits to lumbar concentration data. Green circles = smoothed data. Black circles = raw data.

Supplementary Methods 2: *Estimation of CSF volumes*

Measurement of total CSF volumes by MRI is challenging due to low contrast between bone and CSF.[2] In the current dataset, a trend was observed between the FreeSurfer 'Estimated intracranial volume' and 'total brain volume', with some outliers that approach or achieve negative total cranial CSF volumes (Supplementary Figure 5A; 'total brain volume' is the supratentorial volume + cerebellum cortex and white matter + brain stem) The outliers were removed (Supplementary Figure 5B). We then sought to find a surrogate measure of CSF volumes that was less sensitive to measurement error.

We explored alternative measures of brain morphology that could better correlate with age. The total ventricle volume was converted to a 'ventricle radius' by assuming a spherical shape for the sum of the lateral, third and fourth ventricle volumes. The same was done for the total supratentorial brain volume, producing a 'brain radius'. Assuming that the brain parenchyma 'sphere' perfectly surrounded the ventricle 'sphere', the difference between these two radii yielded a 'brain thickness' (Equation 1, with n = 1/3). This is distinct from MRI-measured cortical thicknesses, due to the inclusion of white matter and basal ganglia.

$$Brain\ thickness = \left(\frac{V_{supratentorial}}{\frac{4}{3}\pi}\right)^n - \left(\frac{V_{ventricle}}{\frac{4}{3}\pi}\right)^n \qquad [1]$$

'Brain thickness' was more highly correlated with age than any MRI-measured value in amyloid positive subjects ($r = -0.54$, $p = 4.8 \times 10^{-4}$, $n = 38$). It was the third highest correlated measure in amyloid negative subjects ($r = -0.64$, $p = 4.9 \times 10^{-8}$, $n = 58$), behind putamen volume and accumbens-area volume (compare to Supplementary Table 4).

To further explore the 'brain thickness' measure, the value of the exponent *n* in equation 1 was varied from 0.001 to 3. The highest correlation with age was found with an exponent of 0.346 for amyloid negative subjects (0.330 when excluding presenilin mutation carriers), and 0.331 for all subjects, supporting the choice of 1/3 for the exponent. This result suggests that brain thinning in normal aging occurs at a more uniform rate over time than volumetric changes.

With outliers removed, the ratio of brain thickness to brain radius was highly-correlated with the total cranial CSF volume (Supplementary Figure 5C; total CSF volume is the total brain volume subtracted from the intracranial volume; $r = -0.71$, $n = 94$, $p = 1.0 \times 10^{-15}$).

The 'brain thickness'/'brain radius' ratio was used to predict cranial CSF volume for all subjects using the regression line in Supplementary Figure 5C. The mean MRI-estimated cranial + cisternal + ventricular CSF volumes were higher (429 ±73 mL) than those in the literature (Alperin et al. : 180 ± 33 mL; Chazen et al. : 179 ± 56 mL).[3,4] All values were scaled so that the average volume of the entire cohort was 180 mL. Although a total CSF amount of 150 mL is widely quoted, this value has been refuted by numerous MRI studies.[3]

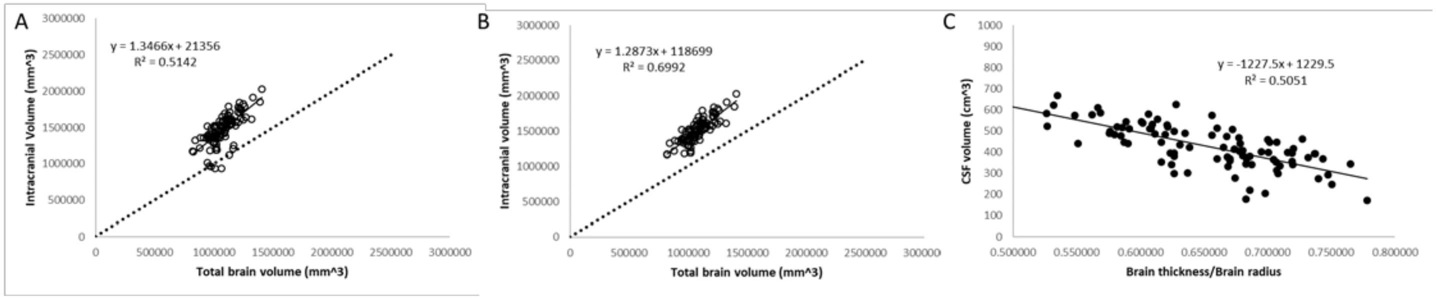

Supplementary Figure 5: A. Intracranial volume estimated from MRI scans exhibited some outliers that showed negligible or negative volumes for the CSF space. B. Outliers were removed. C. The CSF volume was calculated by subtracting the total brain volume from the intracranial volume. Correlation of CSF volume (with outliers removed) was high versus the ratio of brain thickness to brain radius. This ratio accounts for both ventricle enlargement and brain

thinning. The regression line was then used to estimate CSF volume for each subject, based only on their ratio of brain thickness to brain radius.

The CSF volume determined from the linear regression was split between a cranial CSF compartment and the sum of the cisternal volumes. A previous study reported that 16.2% of cranial CSF (excluding ventricles) was in the pontine and cerebelomedullary cisterns.[5] This value was applied uniformly to all subjects.

No information about the subjects' spinal CSF volumes were available in the MRI dataset. An initial value of 80 mL was used for all subjects, which is a value in close agreement with multiple studies, even including elderly subjects.[6–8] These studies report that the correlation between height and CSF volume is weak, so spinal CSF volumes were not corrected for subject height.

Cross-sectional areas of the spinal SAS generally show a constriction near the junction of the cervical and thoracic vertebrae and the junction of the thoracic and lumbar vertebrae.[6,8] The spinal SAS volume was therefore divided into three compartments, roughly corresponding to cervical, thoracic and lumbar volumes. The volume of each compartment was set to 26.9%, 29.5% and 43.6% of the total spinal SAS volume, respectively,[6] which was applied uniformly to all subjects.

Supplementary References

1. Moriyama, E., Ogawa, T., Nishida, A., Ishikawa, S. & Beck, H. Quantitative analysis of radioisotope cisternography in the diagnosis of intracranial hypotension. *J. Neurosurg.* **101**, 421–426 (2004).

2. Eritaia, J. *et al.* An optimized method for estimating intracranial volume from magnetic resonance images. *Magn. Reson. Med.* **44**, 973–977 (2002).

3. Chazen, J. L. *et al.* Automated segmentation of MR imaging to determine normative central nervous system cerebrospinal fluid volumes in healthy volunteers. *Clin. Imaging* **43**, 132–135 (2017).

4. Alperin, N., Bagci, A. M., Lee, S. H. & Lam, B. L. Automated quantitation of spinal CSF volume and measurement of craniospinal CSF redistribution following lumbar withdrawal in idiopathic intracranial hypertension. *Am. J. Neuroradiol.* **37**, 1957–1963 (2016).

5. Bottan, S., Poulikakos, D. & Kurtcuoglu, V. Phantom model of physiologic intracranial pressure and cerebrospinal fluid dynamics. *IEEE Trans. Biomed. Eng.* **59**, 1532–1538 (2012).

6. Sass, L. R. *et al.* A 3D subject-specific model of the spinal subarachnoid space with anatomically realistic ventral and dorsal spinal cord nerve rootlets. *Fluids Barriers CNS* **14**, 1–16 (2017).

7. Alperin, N., Bagci, A. M., Lee, S. H. & Lam, B. L. Automated quantitation of spinal CSF volume and measurement of craniospinal csf redistribution following lumbar withdrawal in idiopathic intracranial hypertension. *Am. J. Neuroradiol.* **37**, 1957–1963 (2016).

8. Edsbagge, M., Starck, G., Zetterberg, H., Ziegelitz, D. & Wikkelso, C. Spinal cerebrospinal fluid volume in healthy elderly individuals. *Clin. Anat.* **24**, 733–740 (2011).
