# Supplementary material for: Importance of CSF-based Aβ clearance with age in humans increases with declining efficacy of blood-brain barrier/proteolytic pathways
Source: Commun Biol. 2022 Jan 27;5:98. doi: 10.1038/s42003-022-03037-0 (PMC8795390; doi:10.1038/s42003-022-03037-0)
Supplement: Supplementary file 2 — Description of Additional Supplementary Files [file 42003_2022_3037_MOESM2_ESM.pdf]

Description of Additional Supplementary Files

File name: Supplementary Data 1

Description: Mass spec and concentration data. Provided as .mat files for use with example program on GitHub (https://zenodo.org/badge/latestdoi/437570053).

File name: Supplementary Data 2

Description: Description of model results. See Supplementary Data 3 for model results.

File name: Supplementary Data 3

Description: Model results. See Supplementary Data 2 for description of parameters and derived values.
