# Supplementary material for: Importance of CSF-based Aβ clearance with age in humans increases with declining efficacy of blood-brain barrier/proteolytic pathways
Source: Commun Biol. 2022 Jan 27;5:98. doi: 10.1038/s42003-022-03037-0 (PMC8795390; doi:10.1038/s42003-022-03037-0)
Supplement: Supplementary file 6 — Reporting Summary [file 42003_2022_3037_MOESM6_ESM.pdf]

nature research

Corresponding author(s): Donald L. Elbert
Last updated by author(s): Nov 24, 2021

Reporting Summary

Nature Research wishes to improve the reproducibility of the work that we publish. This form provides structure for consistency and transparency in reporting. For further information on Nature Research policies, see our Editorial Policies and the Editorial Policy Checklist.

Statistics

For all statistical analyses, confirm that the following items are present in the figure legend, table legend, main text, or Methods section.

n/a	Confirmed	
☐	☒	The exact sample size (n) for each experimental group/condition, given as a discrete number and unit of measurement
☐	☒	A statement on whether measurements were taken from distinct samples or whether the same sample was measured repeatedly
☐	☒	The statistical test(s) used AND whether they are one- or two-sided<br>*Only common tests should be described solely by name; describe more complex techniques in the Methods section.*
☐	☒	A description of all covariates tested
☐	☒	A description of any assumptions or corrections, such as tests of normality and adjustment for multiple comparisons
☐	☒	A full description of the statistical parameters including central tendency (e.g. means) or other basic estimates (e.g. regression coefficient) AND variation (e.g. standard deviation) or associated estimates of uncertainty (e.g. confidence intervals)
☐	☒	For null hypothesis testing, the test statistic (e.g. F, t, r) with confidence intervals, effect sizes, degrees of freedom and P value noted<br>*Give P values as exact values whenever suitable.*
☒	☐	For Bayesian analysis, information on the choice of priors and Markov chain Monte Carlo settings
☒	☐	For hierarchical and complex designs, identification of the appropriate level for tests and full reporting of outcomes
☐	☒	Estimates of effect sizes (e.g. Cohen's d, Pearson's r), indicating how they were calculated

Our web collection on statistics for biologists contains articles on many of the points above.

Software and code

Policy information about availability of computer code

Data collection	Freesurfer 5.3, MassLynx V4.1, Xcalibur V2.1
Data analysis	Julia 1.4.1 (CSV v0.7.7, DataFrames v0.21.5, DifferentialEquations v6.15.0, MAT v0.8.0, NLsolve v4.4.0, Optim v0.22.0, Plots v1.5.8), MATLAB 2020a, RStudio 1.3.1073, custom scripts in Julia, MATLAB and R.

For manuscripts utilizing custom algorithms or software that are central to the research but not yet described in published literature, software must be made available to editors and reviewers. We strongly encourage code deposition in a community repository (e.g. GitHub). See the Nature Research guidelines for submitting code & software for further information.

Data

Policy information about availability of data

All manuscripts must include a data availability statement. This statement should provide the following information, where applicable:

- Accession codes, unique identifiers, or web links for publicly available datasets
- A list of figures that have associated raw data
- A description of any restrictions on data availability

Data requests may be made to RJB (SILK & concentration) and TLSB (MRI), by qualified researchers capable of maintaining subject privacy. De-identified model results are included as supplemental data. De-identified SILK and concentration data are available as supplemental data.

Field-specific reporting

Please select the one below that is the best fit for your research. If you are not sure, read the appropriate sections before making your selection.

- [x] Life sciences
- [ ] Behavioural & social sciences
- [ ] Ecological, evolutionary & environmental sciences

For a reference copy of the document with all sections, see nature.com/documents/nr-reporting-summary-flat.pdf

Life sciences study design

All studies must disclose on these points even when the disclosure is negative.

Sample size	Sample sizes (n = 100) were determined based on mass spec sensitivity to be sufficiently powered to detect differences in Abeta kinetics between AD and non-AD.
Data exclusions	As described in the manuscript, four subjects were excluded due to poor fits to SILK data (n = 2) or concentration data (n = 2). Data quality was poor in these subjects, making it difficult to judge the quality of the fits.
Replication	This is a retrospective study of experiments that were performed between 2006-2012. No study of this kind and magnitude has been undertaken in the intervening time.
Randomization	All subjects underwent the same experimental protocols.
Blinding	Those with direct contact with the subjects were blinded to amyloid and mutation status.

Reporting for specific materials, systems and methods

We require information from authors about some types of materials, experimental systems and methods used in many studies. Here, indicate whether each material, system or method listed is relevant to your study. If you are not sure if a list item applies to your research, read the appropriate section before selecting a response.

Materials & experimental systems

n/a	Involved in the study	
x		Antibodies
x		Eukaryotic cell lines
x		Palaeontology and archaeology
x		Animals and other organisms
	x	Human research participants
x		Clinical data
x		Dual use research of concern

Methods

n/a	Involved in the study	
x		ChIP-seq
x		Flow cytometry
x		MRI-based neuroimaging

Human research participants

Policy information about studies involving human research participants

Population characteristics	Subjects were recruited for two trials. The LOAD study consisted of subjects 60-88 years of age. The FACS study enlisted participants who were siblings. The age of onset for the mutations varied, and age of participants spanned from 29-72 years of age. The data sets were combined to provide a broad age range. Experimental protocols between the studies were identical, except that each project had a dedicated mass spec instrument.
Recruitment	Subjects were recruited through the Alzheimer's Disease Research Center at Washington U. in St. Louis. The implicit bias is that the subjects who participated likely had a familial, social or professional experience with Alzheimer's Disease. However, diversity in race and gender was actively sought.
Ethics oversight	The studies were approved by the Washington University Human Studies Committee and the General Clinical Research Center Advisory Committee. All participants completed informed written consent.

Note that full information on the approval of the study protocol must also be provided in the manuscript.
